# Viruses of Eukaryotic Algae: Diversity, Methods for Detection, and Future Directions

**DOI:** 10.3390/v10090487

**Published:** 2018-09-11

**Authors:** Samantha R. Coy, Eric R. Gann, Helena L. Pound, Steven M. Short, Steven W. Wilhelm

**Affiliations:** 1 The Department of Microbiology, The University of Tennessee, Knoxville, TN 37996, USA; srose16@tennessee.edu (S.R.C.); egann@tennessee.edu (E.R.G.); hpound@tennessee.edu (H.L.P.); 2 The Department of Biology, The University of Toronto Mississauga, Mississauga, ON L5L 1C6, Canada; steven.short@utoronto.ca

**Keywords:** eukaryotic algal virus, algal-NCLDV, Picornavirales, phytoplankton

## Abstract

The scope for ecological studies of eukaryotic algal viruses has greatly improved with the development of molecular and bioinformatic approaches that do not require algal cultures. Here, we review the history and perceived future opportunities for research on eukaryotic algal viruses. We begin with a summary of the 65 eukaryotic algal viruses that are presently in culture collections, with emphasis on shared evolutionary traits (e.g., conserved core genes) of each known viral type. We then describe how core genes have been used to enable molecular detection of viruses in the environment, ranging from PCR-based amplification to community scale “-omics” approaches. Special attention is given to recent studies that have employed network-analyses of -omics data to predict virus-host relationships, from which a general bioinformatics pipeline is described for this type of approach. Finally, we conclude with acknowledgement of how the field of aquatic virology is adapting to these advances, and highlight the need to properly characterize new virus-host systems that may be isolated using preliminary molecular surveys. Researchers can approach this work using lessons learned from the *Chlorella* virus system, which is not only the best characterized algal-virus system, but is also responsible for much of the foundation in the field of aquatic virology.

## 1. Introduction

Viruses infecting eukaryotic algae are extremely diverse. They have been reported with DNA or RNA genomes in various architectures (linear, circular, double-stranded, single-stranded, segmented) and sizes (4.4 to 638kb) [1]. Some viruses accomplish infection with just a few viral genes at their disposal, while others maintain a gene arsenal nearly 100 times that size. Viruses infecting algae influence large ecological and biogeochemical processes. They direct the evolution of hosts through predator-prey selection and genetic exchange, consequently influencing algal fitness, population dynamics, and ultimately, microbial community structure. Infection can also alter the composition and distribution of organic matter in the environment (a process referred to as the aquatic ”viral shunt” [2]) and influence particle size-distribution, nutrient cycling, and biological system activity (e.g., respiration [3]). While algal viruses are important members in many aquatic environments, their contribution to these processes at the global scale primarily arises when they infect and lyse abundant bloom-forming algae. This includes harmful bloom formers and ecosystem scale specialists like coccolithophores that form blooms large enough to be observed from outer space [4].

**Figure 1:**
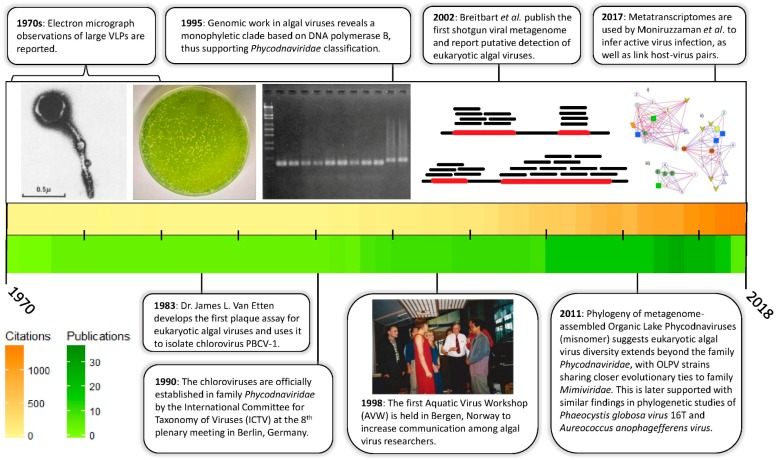
Timeline of eukaryotic algal virus research with important milestones highlighted. Colored bars represent the annual citations and publications generated from a Web of Science Citation Report using the field tag TS = (algal virus) for all databases. The search was conducted on 8 May 2018 at 11:00 a.m. Citation Report results were visualized as heatmaps using custom R scripts. Electron micrograph image [10] and electrophoretic gel [11] reprinted by permission. Network analysis [12] reprinted under authority of Creative Commons.

It is a relatively recent realization that algal viruses are ecologically significant. In fact, the whole history of algal virus research has occurred primarily in just the last half century (Figure 1). While there have been sporadic observations of virus infection of algae cultures since the early 1970s [5,6], the importance of algal viruses in natural systems was brought into the limelight by a series of observations of virus-like-particles associated with important bloom-forming algae [7,8,9]. These findings inspired questions about the identity and evolutionary relationships within these virus-host systems. Such questions, however, required viruses to be isolated and genetically characterized.

One of the first algal-virus systems to achieve “model” status were the double-stranded DNA (dsDNA) viruses that infect the unicellular, ex-symbiotic, green alga *Chlorella* [13]. The *Chlorella* virus-host model system remains the best characterized of all the algae-virus models, with genomes [14,15,16,17,18], transcriptomes [19,20], and proteomes [14] documented in the literature. Indeed, it was sequencing of the DNA polymerase B (*polB*) genes from *Chlorella* viruses PBCV-1 and NY-2A [21], and later from *Micromonas pusilla virus* SP1, that revealed a conserved amino acid sequence distinct from other known polB protein sequences. This observation enabled the development of degenerate PCR primers that selectively amplified these algal-virus *polB* genes [11,22]. The sequences of these PCR amplicons supported a unique monophyletic viral clade, now recognized as the family *Phycodnaviridae* of the Nucleocytoplasmic Large dsDNA Viruses (NCLDV). For a while the *Phycodnaviridae* was thought to be home to all of the large dsDNA algal viruses: perhaps even dominating the overall algal virus community. This perspective changed when sequencing of new isolates demonstrated that their “core” genes were more closely related to genes from the protist-infecting “giant viruses” of family *Mimiviridae* [23,24,25]. In general, algal-infecting viruses are recognized as members of one of these two families, though future work may challenge the monophyletic nature of these groups. For example, clustering of the *Phycodnaviridae* is at times disrupted when homologs from other cellular or viral families are included in phylogenetic reconstructions [26,27,28]

There have also been increasing reports of single-stranded DNA (ssDNA) viruses, mostly infecting diatoms, RNA viruses (Table 1) [29,30], and even parasites of these large algal viruses known as virophage [31,32]. The most informative reports on these systems have come from metagenomic and metatranscriptomic datasets that can detect the presence and activity of a wide range of DNA and RNA viruses. In turn, the known diversity of eukaryotic algal viruses has greatly expanded, at times even yielding putative full-length viral genome assemblies [12]. Perhaps most promising is the possibility of predicting virus-host relationships in silico [12,33,34], whereas traditional methods have relied on virus isolation from a relative few cultivated algae. Shotgun -omics further create the opportunity to identify virus-host pairs from environmental data and place them in semi-quantitative ecological context. Indeed, these studies may even serve as preliminary assessments of the future cultivation requirements for isolating new virus-host systems. This burgeoning scientific frontier necessitates a review on the known diversity of eukaryotic algal viruses, the molecular toolkit available for in situ studies on their ecology, and the direction aquatic virology is taking to adapt to these methodologies.

## 2. Diversity of Cultured Virus-Host Systems

**Figure 2:**
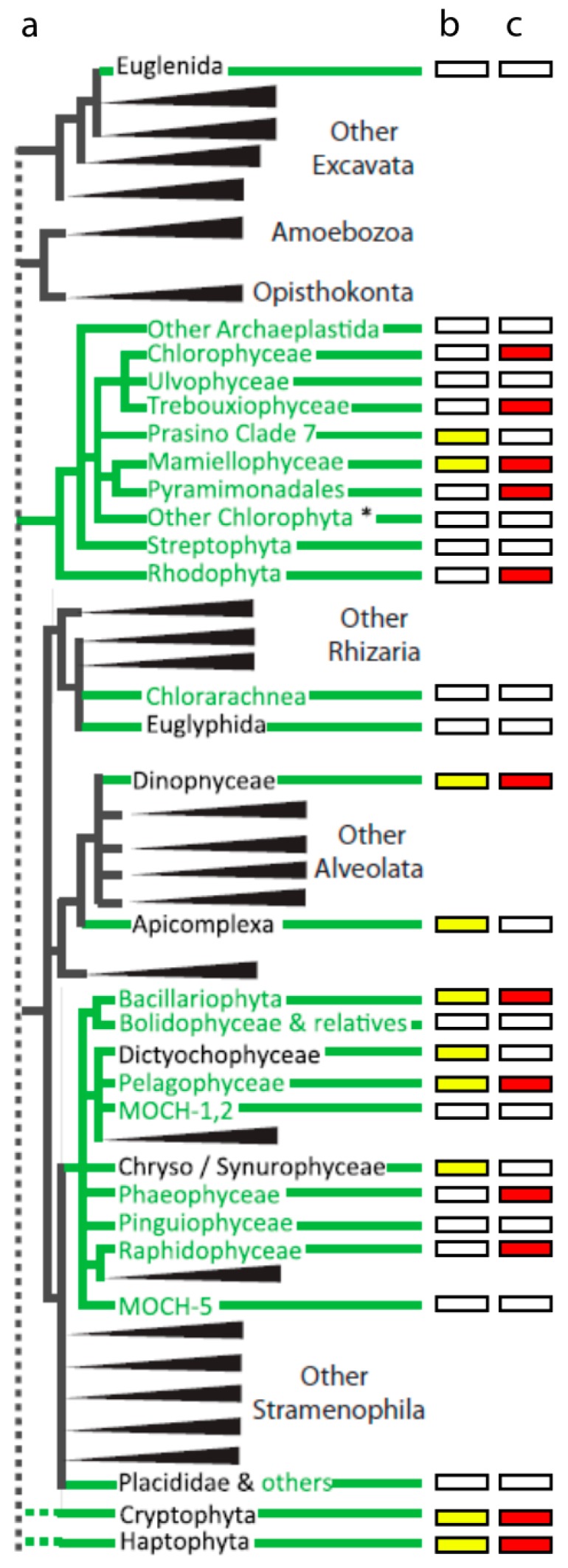
(**a**) Schematic phylogeny adapted from de Vargas et al. demonstrating known virus-interactions with eukaryotic alga lineages. The phylogeny was originally constructed on recognized eukaryotic plankton lineages that were detected in TARA Oceans datasets, which included hits to all aquatic algal containing lineages. We collapsed the original tree to highlight these lineages in the context of their current phylogenetic placement. Green lines denote lineages with photosynthetic algal representatives, whereas the text color indicates whether all or only some representatives are phototrophic-green or black text, respectively; (**b**) Yellow boxes denote the top ten most abundant, planktonic, phototroph-associated lineages based on 18S rDNA surveyed in the TARA Oceans study. Asterisks denote lineages that were artificially grouped for simplicity, and their full descriptions can be found at http://taraoceans.sb-roscoff.fr/EukDiv/; (**c**) Red boxes denote algal-lineages that have an isolated algae-infecting virus in culture collection, though these are not all marine systems. The virus isolates are listed in Table 1.

The diversity of algal viruses mirrors that of their hosts, bearing in mind that the name “algae” does not denote a common evolutionary relationship. Indeed, algae have been observed in freshwater, marine, and terrestrial systems, in unicellular, colonial, or multicellular forms, and in disparate taxonomic lineages. Nevertheless, the diversity of algae can be depicted using an existing taxonomic framework that includes seven “supergroups” consisting of Excavata, Amoebozoa, Opisthokonta, Archaeplastida, the SAR group (Stramenophila, Alveolata, and Rhizaria), and a series of non-delineated, “cryptic” organisms collectively referred to as the *Incerta sedis* [35]. Beyond this framework, the manner in which certain taxa are placed within eukaryotic phylogeny varies in the literature and is a subject of ongoing scientific debate. We adapted the schematic phylogeny presented by the TARA Oceans group [36] to illustrate the diversity of marine eukaryotic plankton, their relative abundance based on TARA Oceans 18S rDNA gene surveys, and lineage association with viruses that have been isolated and are maintained in lab cultures (Figure 2 and Table 1). This framework demonstrates that marine eukaryotic algae are known to occupy all but the Amoebozoa and Opisthokonta supergroups. Algae-infecting viruses have been isolated using hosts spanning almost all abundant planktonic lineages, though many are single systems or instances without genomic information to define viral phylogenetic placement (e.g., TampV). Although *Pyramimonadales* and *Raphidophyceae* were not abundant in the TARA Oceans 18S dataset, select species in these groups are known bloom-formers [37,38,39,40] making the available algal-virus system for these lineages ecologically informative. Viruses have also been isolated on important non-planktonic species, such as brown and red macroalgae (*Phaeophyceae* and *Rhodophyceae*). Abundant lineages without an algae-infecting virus include photosynthetic *Dictyochophyceae*, the Prasino Clade 7 group, the Chryso/Synuro group, and the Apicomplexans—though some of the highly represented lineages could be attributed to non-photosynthetic members. Establishing well characterized host-virus systems in these lineages could be very useful for bloom-forming algae of these lineages. For example, it would be appealing to isolate a *Pseudochattonella* (*Dictyochophyceae*) infecting virus, as the host alga is responsible for fish kills. In 2016 *Pseudochattonella* was responsible for a massive fish kill in Peru amounting to an economic loss of ~$800 million dollars [41]. In another interesting, albeit more complicated example, survival of the red-tide, bloom-forming ciliate *Mesodinium rubrum* depends on ingestion of photosynthetic cryptophytes to obtain necessary organelles (e.g., plastid, mitochondria, nucleus) [42]. Viruses infecting cryptophyte prey may compete with this grazer, thus serving as an important control on the frequency and duration of red tides. Such broad trophic effects have been shown in studies on *Emiliania huxleyi,* where viral-infected cells are ingested by zooplankton at different rates than non-infected cells [43,44].

Eukaryotic algal viruses in culture collections have been isolated from ~60 alga species (Table 1). Most of these are lytic, dsDNA viruses of the NCLDV group with a narrow, known host-range. The abundance of NCLDVs would imply that these are an ecologically relevant algal-virus type in the virus community, but whether or not these are the dominating type is unclear. This would certainly contrast with plant viromes which are dominated by RNA viruses. It is also possible that NCLDVs are more easily detected and isolated, thus explaining why only dsDNA viruses have been isolated from water samples that putatively contained other types of viruses. For example, electron micrographs of bloom-associated *Emiliania huxleyi* cells have been observed to simultaneously contain both small (50–60 nm) and large (185–200 nm) intracellular VLPs [93]. Similar observations been made in *Pyramimonas orientalis* [94], but currently only one type of dsDNA virus has been isolated for this algae [54]. It is possible that these viruses compete for algal infection, but they may also represent a case of virus-infecting virophage that are already known to co-occur with *Mimiviridae* [95,96], and perhaps even *Phycodnaviridae* [31,33] viruses. Observations of co-occurring viruses are not limited to microscopy either; network analysis of metatranscriptomic data has linked the brown alga *Aureococcus anophagefferens* to its known dsDNA virus AaV as well as to uncharacterized ssDNA viruses [12], although the mechanism of this linkage (either direct, or via a co-occurring microbial host of the virus) remains elusive. In short, algae may be infected by many types of viruses, potentially at the same time, and the numerically dominant virus type may not always represent that which is in the culture collection.

To date, there are four algal species that are known to be infected by diverse viruses comprised of different nucleic acid types. These include *Heterosigma akashiwo, Chaetoceros tenuissimus, Micromonas pusilla,* and *Heterocapsa circularisquama,* and in all cases the different virus types infect the same host strain [97]. The coexistence of *Heterosigma akashiwo* viruses HaRNAV and HaDNAV is especially intriguing given these viruses exhibit opposite infection dynamics; the RNA virus has a high viral production rate, but a slower lytic cycle, whereas the DNA virus quickly replicates but produces fewer particles [81]. It was hypothesized that coexistence could be maintained through variable host densities and viral decay rates, thus representing viruses that may have evolved as r- or k- strategists as has been proposed for *Heterocapsa* viruses [98], but is certainly not supported enough to be extrapolated as an explanation for all co-occurring viruses. Even virus isolates of the same nucleic acid type and species can exhibit considerable diversity. This can be extreme in some cases, where dsDNA viruses infecting the same algal host, which would be expected to cluster phylogenetically, are affiliated with NCLDV viral families *Mimiviridae* or *Phycodnaviridae* (e.g., *Phaeocystis globosa* Virus Groups I and Groups II [25,99]. It is possible that eukaryotic algae may commonly be infected by viruses of diverse replication strategies, and evolutionary histories, but the extent of this, as well as the factors that may allow this, needs more thorough investigation.

### 2.1. dsDNA Viruses Infecting Eukaryotic Algae

Most dsDNA viruses infecting algae are members of the NCLDV group, with the proposed exception of Tsv-N1 [46]. Algal-NCLDV viruses have large genomes that encode hundreds of protein coding genes. Their evolutionary relationship has been inferred by core genes conserved across NCLDVs [100], placing them into either the family *Phycodnaviridae* or as extended members of the family *Mimiviridae*. Algal viruses of the latter group have recently been given the proposed distinction of Mesomimivirinae [101], but for our purposes we will maintain the *Mimiviridae* description. The one exception to these two family assignments is HcDNAV, which shares closer similarity to the family *Asfarviridae* [56]. To date, the NCLDV core gene compliment has been reduced to just a few genes (e.g., D5R packaging ATPase, D13L major capsid protein, and B family DNA polymerase), implying that the genetic diversity is huge among this group. Indeed, a genomic comparison among *Phycodnaviridae* members PBCV-1 (Chloroviruses), EsV-1 (Phaeoviruses), and EhV-86 (Coccolithoviruses) yielded only 14 conserved homologs from a pool of ~1000 genes [102]. A more comprehensive look at these diverse genes can be found in genus-specific reviews of the *Phycodnaviridae* [17,47,51,103,104,105]. 

It is anticipated that any single algal host can be permissive to many closely related virus variants, whereby phylogenetic comparisons of their core genes will reveal distinct clades (e.g., *Micromonas pusilla* and *Chlorella variabilis* viruses) with differences in latent phases, burst sizes, and genome size [17]. In closely related viruses this is best resolved using concatenated alignments of marker protein sequences. At the same time, the origin of some of these genes is often attributed to gene transfer events. Many algal NCLDVs have acquired non-ancestral genes, but the majority of these appear to come from difference sources: Prasinoviruses acquire most of these from their host, Chlorovirus non-ancestral genes mostly derive from bacteria [106], and *Aureococcus anophagefferens* Virus (AaV) encodes a more even mixture of host, bacterial, archaeal, and viral genes [23]. At the same time, it is worth noting that the origin of some genes could be difficult to ascertain if only a limited subset of viral (and host) homologs have been sequenced and annotated in public databases. Regardless, it has been suggested that viruses whose hosts are in closer association with bacteria tend to encode more putative non-ancestral genes, and that these genes cluster near the terminal ends of the viral genome [107]. However, while the *Chlorella* algae is an endosymbiont of *Paramecium* that is certainly in close proximity to bacteria, the non-ancestral genes carried by the virus are evenly dispersed across its genome [17]. In contrast, AaV displays terminal clusters of non-ancestral genes [23], but its host is a free-living photo/osmotroph. In either case, the biological implication of such high viral gene diversity, and how it is generated, is unclear. It may help the virus acquire its specific needs for infection but has also been proposed to allow viruses to infect multiple hosts.

### 2.2. ssDNA Viruses Infecting Eukaryotic Algae

To date, the only ssDNA alga-infecting viruses that have been isolated are those which infect diatoms (Bacillariophyceae). In total, diatoms are a collective of an estimated 12,000–30,000 species, representing one of the most abundant phytoplankton groups in freshwater and marine environments [108]. Most diatom-virus systems currently in culture are those infecting the cosmopolitan genus *Chaetoceros*. These isometric virus particles are ~35 nm in diameter and house circular, ssDNA genomes ranging from ~5.5–6.0 kb [66]. The genomes generally encode four open reading frames consisting of an endonuclease (Rep), a major capsid protein, and two ORFs with unknown function. The capsid and replication initiating endonuclease are used in phylogenetic analyses. Three new members (whose genomes are ~4.5–4.7 kb) were recently reported from a de novo assembly of metagenomic reads from the mollusk *Amphibola crenata* and from sediment within an estuary in New Zealand [109]. Phylogenetic analysis of the capsid proteins suggest this gene is a recent acquisition from ssRNA viruses, which is interesting, though not without precedent [110,111]. These metagenome assembled viruses have resulted in the taxonomic reclassification of diatom viruses into the family *Bacilladnaviridae* that includes cultured diatom viruses noted in Table 1 with asterisks [112]. Many other ssDNA viruses are being detected in omics datasets [12], though resolving their specific host is an ongoing challenge.

### 2.3. RNA Viruses Infecting Eukaryotic Algae

Algae-infecting viruses with single (ss) and double-stranded (ds) RNA genomes have also been isolated and characterized, although most attention has been focused on the ssRNA isolates. Both virus groups encode an RNA-dependent RNA polymerase (RdRP), as well as proteases and helicases that can be used to infer distant evolutionary relationships. Most information on dsRNA algal viruses has been derived from the original isolation papers describing the evolutionary relationships of the isolates. MpRV, a dsRNA virus of *Micromonas pusilla*, forms its own genus within the family *Reoviridae* (unassigned order) and has been proposed to be the ancestral line of the *Reoviridae* based on its placement between clades that demonstrate turreted or non-turreted virions [113]. The other dsRNA virus isolate is *Chondrus crispus* virus (CcV), a toti-virus like entity. CcV represents an extraordinary case of a putative quasispecies virus that was accidentally discovered when a small band of dsRNA (~6 kb) was observed during host genomic preparation for sequencing [55]. Similar dsRNA bands have been observed in extracts from all algal life phases, geographic locations, and in extracts from other red algae, though virus-like-particles and host lysis was not observed. The CcV system may represent either a latent or chronic (i.e., particle production below the limit of detection) viral infection that is ubiquitous among red algae, similar to known latent dsDNA viral infections of brown algae by Phaeoviruses [114]. Since both *Chondrus crispus* and *Micromonas pusilla* are ecologically important algae, characterization of their relationship with these viruses is important and perhaps reflective of a need to search for more dsRNA viruses associated with algae.

**Figure 3:**
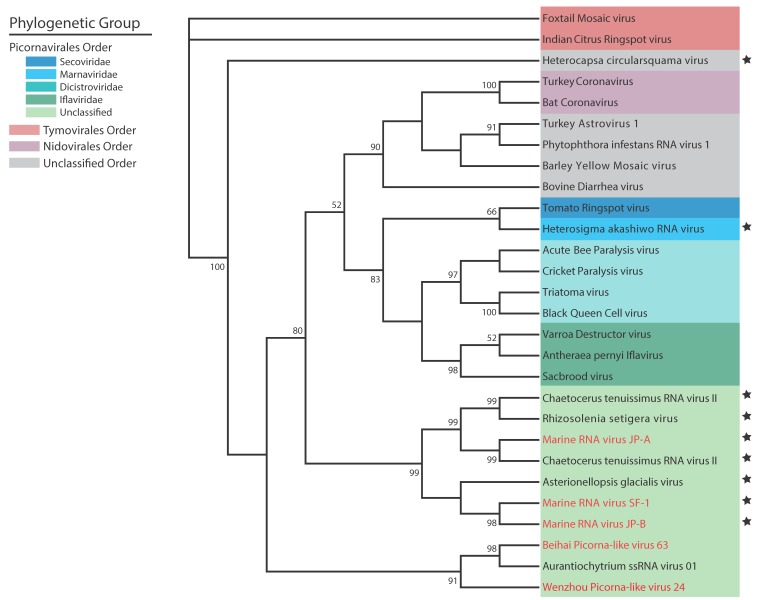
Diversity of single-stranded RNA viruses depicted based on phylogeny of RNA-dependent RNA polymerase (Rdrp NCBI CDD:01699) reference sequences downloaded from NCBI RefSeq database (see in Supplemental Table S1). Sequences were aligned and trimmed in Mega7 [117] and an unrooted maximum likelihood phylogeny was created using PhyML 3.0 with LG model [118]. Empirical equilibrium frequencies were used with aLRT SH-like statistics for branch support. Phylogenetic groups are color coded with algal viruses denoted by a star. Viral isolates from metagenomic assemblies are in red text.

ssRNA viruses have received considerably more attention since their hosts are common marine phytoplankton with some species capable of forming harmful blooms [39,115,116]. Most of the alga-infecting ssRNA viruses are members of the order Picornavirales (Figure 3), with a few contradictions that are awaiting a taxonomic re-evaluation based on molecular data. The viruses infecting *Heterocapsa* and *Heterosigma* are the sole members of the families *Alvernaviridae* (unassigned order) and *Marnavirdiae* (order Picornavirales), respectively [109,112], while the genus *Bacillarnavirus* (order Picornavirales) includes formal members *Chaetoceros socialis forma radians* RNA virus, *Chaetoceros tenuissimus* RNA virus 01, and *Rhizosolenia setigera* RNA virus 01. Other diatom viruses Csp03RNAV, AglaRNAV, and CtenRNAV type II are putative members of *Bacillarnavirus* based on phylogenetic relationships of replicase or structural proteins [1]. The diatom viruses are generally thought to be highly species specific based on host-range experiments, with the exception of CtenRNAV type II which can infect four *Chaetoceros* sp. in addition to *Chaetoceros tenuissimus* [66]. These viruses and their hosts represent ecologically important systems that may reveal much on the persistence, co-existence, and competition of diatom viruses.

## 3. Culture Independent Approaches: Expanding Known Diversity

### 3.1. PCR Applications for Estimating Viral Diversity and Dynamics

**Figure 4:**
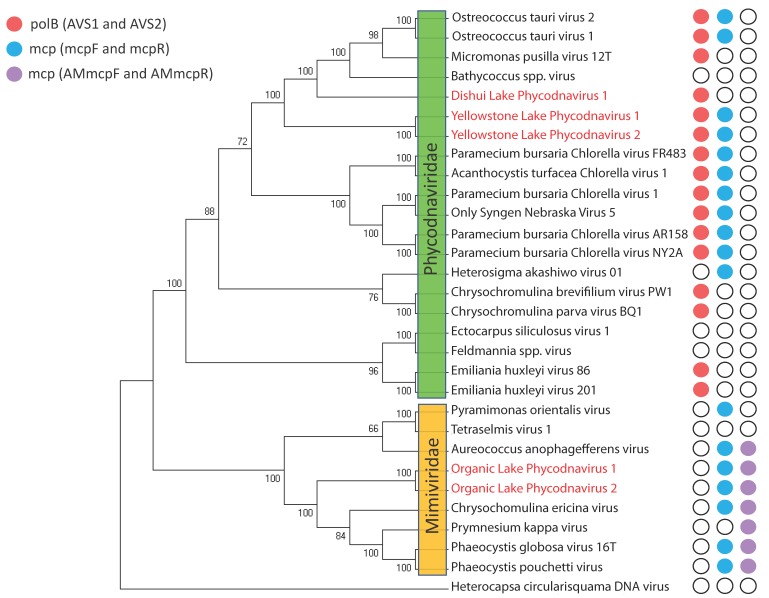
Phylogenetic tree depicting the evolutionary relationships of algal NCLDVs based on amino acid alignment (ClustalW) of the core gene, DNA polymerase B (see in Supplemental Table S2). The tree was built using the maximum likelihood method based on the JTT matrix-based model with 200 iterations in MEGA7 [117]. Viruses belong either to the family *Phycodnaviridae* or are recognized “extended members” of the family *Mimiviridae*. The recently discovered dinoflagellate infecting virus, *Heterocapsa circularisquama* DNA virus, was used to root the tree and shows little similarity to other algal NCLDVs despite being a large DNA virus. Viruses in red text denote metagenome assembled viral genomes, meaning their association with an alga host is putative. Colored dots to the right indicate the viruses can be putatively PCR amplified by the respective PCR primer set based on ≥90% match between each primer and its respective target binding site. This equates to ≤2 primer mismatches, which has been shown to be capable of producing a PCR reaction, albeit at lower efficiency (for RT-qPCR) [126]. The same study shows that three or more mismatches in the same primer completely inhibit a PCR reaction, and is an observation that aligns with failed PCR reactions reported for *Ectocarpus siliculosus* virus 1 and *Feldmannia* spp. virus [11]. PCR amplification predictions were done using motif searches in CLC Genomics and the software De-MetaST-BLAST [127].

Developing algal-virus model systems in the lab can inform much on the biology and ecology of algal viruses, but dependence on these systems is a limiting step. The ability to determine viral geographic distributions, population fluctuations, and diversity ultimately depends on analysis of environmental samples. Microscopic methods [119], flow cytometry [120,121,122], and infectivity assays (e.g., most probable number, plaque assay [13]) have been used to answer these questions, but these approaches lack taxonomic resolution and/or the relatively quick processing time that molecular techniques provide. To date, the principal molecular method for studying environmental algal viruses has been based on PCR amplification of conserved marker genes. Most of this work has focused on algal NCLDVs using polB [11] and the NCLDV major capsid protein (mcp) as gene targets [123]: subsets of this community have been further examined using primers that specifically target the extended, algal *Mimiviridae* major capsid protein (AMmcp) [124]. For reference, the potential amplification ranges of these primers are mapped against a phylogeny of sequenced virus isolates (Figure 4). There has been discussion on amplification bias of *polB* primers based on observations that environmental datasets tend to amplify prasinoviruses, even though these may be environmentally abundant viral types [98]. The gene amplified by this primer set has also been suggested to be a poor marker for resolving within algal virus genera. For example, there are two distinct groups of *Phaeocystis globosa* infecting viruses, and these groups phylogenetically cluster into different families [1]. Diversity may be better assessed using genome fluidity measurements of the pan-genome [125], but this would work better for describing viruses with full-genome sequences. Indeed, marker gene primer sets remain useful for elucidating environmental diversity of algal NCLDVs.

A recent clone library of PCR amplicons generated using the two mcp primer sets demonstrates a wide diversity of algal viruses isolated from marine and freshwater environments [124]. This study also used PCR amplification to track the occurrence and dynamics of virus groups (defined by sequence clustering as operational taxonomic units, OTUs) over the course of a harmful brown-alga event. Biases aside, the approach used in that study has certainly expanded the known diversity of algal NCLDVs. It has also shown that cultured viral isolates are often distinct from environmental viruses, and that viruses are widely dispersed in the environment [123,124,128,129,130,131]. Another recent group of primer sets was developed by Wilson et al. that amplifies a putative algal-*Mimiviridae* specific mismatch repair gene (*MutS*) [132]. Novel groups of algal NCLDVs were detected in all of the samples tested, making this gene/primer set another potentially useful tool for studying virus diversity. RNA virus diversity has been assessed using primer sets targeting RNA dependent RNA polymerase (RdRP), a protein encoded by all RNA viruses [29,133]. This led to the discovery of a highly diverse super group of putative, marine, protist-infecting picorna-like viruses [133] that are consistently represented in metagenomic datasets [134]. Moreover, alignments of conserved regions of RdRP form clades that are congruent with virion structure, host, and epidemiology [29].

While diversity can be addressed with degenerate primer PCR amplification, one of the major drawbacks of this approach is that it is generally not suitable for quantitative measurements [135]. Indeed, degeneracies allow for biases in primer-binding and template amplification in mixed communities [136]. Use of more specific primer sets and quantitative PCR approaches can avoid this issue [137,138], but at the risk of not detecting closely related viruses. Even when using specific primer sets, recent duplications of marker genes can result in overestimation of viral abundances. One of the recent developments to overcome this is to spatially separate viruses and subject them to solid-phase, single-molecule PCR polony amplification [139]. Family specific degenerate primers amplify diverse members without the issue of competitive amplification, then categorize and quantify the amplicons using probes for virus group specific genes. Of course, this method is also dependent on prior sequence knowledge on the virus types of interest and has been validated only in cyanophage thus far, but it is certainly an appealing method for the study of eukaryotic alga infecting viruses. Another recently discovered application of PCR is its potential to link viruses and hosts. Microfluidics can be used to isolate infected single-cells that can then be subjected to simultaneous PCR detection of viral and host genes [140].

### 3.2. Using Omics Approaches to Estimate Virus Diversity and Dynamics

Because community scale genomics and transcriptomics are not dependent on target amplification, they are better suited for resolving viral diversity and can in some cases allow for the assembly of complete viral genomes. Though this is more readily accomplished in small RNA and DNA viruses [12,109,141], it has also been possible for some large dsDNA viruses and virophage [24,31,32]. This potential is so valuable that a proposal was recently submitted to the International Committee on the Taxonomy of Viruses (ICTV) for the inclusion of metagenomic-assembled viruses into the official classification scheme [142]. Not only was this approved, but it initiated a change in the primary approach ICTV uses for virus classification from phenotypic characterization based on viral isolates to molecular characterization based on viral DNA sequences. Since this time, metagenome assembled circular Rep-encoding single-stranded (CRESS) DNA viruses have been properly classified, including the *Bacilladnaviridae* [109], the putative vertebrate infecting *Smacoviridae* [143], and many more [112,144]. Some of the initial taxonomic classifications may also need to be reassessed in light of molecular methods, as classical taxonomy based on phenotype is not always congruent with phylogenetic clustering: The order Nidovirales may in fact belong to the Picornavirales.

While becoming more common, sequencing entire viral communities remains challenging and each experimental step must be considered in the context of existing biases and the project objectives. Virus particles have very low nucleic acid contents, necessitating amplification, concentration, or enrichment to obtain adequate sequencing depth. Simple approaches to do this involve concentration of environmental samples via filtration [145] or chemical flocculation [146]. Virus enrichment can be done for specific viral types with some quantitative applications. For example, dsDNA can be quantitatively amplified using fusion PCR primers, and adaptase will quantitatively amplify both ssDNA and dsDNA viruses [147]. Rolling circle amplification can increase detection of circular viruses [109], and recombinant plant proteins that non-specifically bind dsRNA can select for dsRNA viruses [148]. There are also methods to separate DNA and RNA viruses for separate analyses using hydroxyapatite-mediated techniques [149]. One of the most appealing enrichment strategies recently used involves selection (*via* binding) of poly-A containing nucleic acid (i.e., mRNA) to focus on the active viral community [12]. This is a useful signal to distinguish virus particles from active infection, as the former will not produce an mRNA signal, though this excludes some (+) ssRNA viruses that have polyadenylated genomes independent of infection [150]. Though all of these methods are useful for improving detection, there are biases to be considered before making conclusions about viral abundances. These issues have been elucidated for sampling, extraction, and purification methods [151,152], but these studies are not comprehensive.

The viral sequences generated from any sequencing approach are subjected to a general analytical workflow involving quality filtering, assembly, annotation, and diversity analyses. Many tools are available to perform this bioinformatic workflow [153], but few of these are designed to complete the full workflow. Moreover, careful understanding of the sequence databases searched in each workflow is necessary to know whether biases exist for particular virus types. GenBank and the nt/nr databases are preferred as these are continually updated and contain information for all virus types; however, their large size can slow processing considerably. To overcome this, creating custom workflows using marker genes of interest can speed up processing time while maintaining the ability to detect diverse virus types.

**Figure 5:**
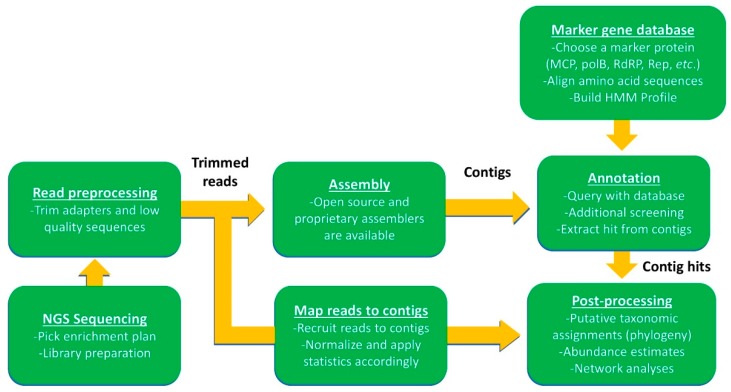
General bioinformatic pipeline using marker gene probing of community sequence data. This framework follows that used by Moniruzzaman et al., 2017 [12], where viral activity was assessed using marker gene detection from environmental mRNA. Though this framework was modeled off the cited study, it is flexible enough to incorporate both metagenomic and metatranscriptomic applications.

An example of a bioinformatics workflow using a custom marker gene database to interpret NGS sequences (i.e., Illumina™ paired-end sequencing) is shown in Figure 5. First, reads must be preprocessed to remove contaminating adapter sequences and trim low-quality reads. The next step involves assembly of reads into larger contigs, followed by contig annotation using a database of known sequences and a homology or alignment search tool (BLAST, HMMER, Bowtie2, etc.). BLAST tools have commonly been used for this purpose in cellular organisms, and even in some virus studies [33], but may be less efficient for identifying novel virus homologs since they often have low pairwise sequence identities [154]. An alternative to using sequence alignments are Hidden Markov models (MMS), which score hits to protein domains. These analyses can be done with the search tool HMMER to create a marker gene database (HMM-build) that can be queried against assembled contigs [155]. Once viral contigs have been identified, the relevant gene hits can be extracted for post-processing (i.e., phylogenetic analysis). In many cases, especially when using small databases, it is useful to verify viral hits with a second similarity search of the extracted gene. Following verification, extracted viral hits can be placed unto an existing phylogenetic tree built with homologous reference sequences (e.g., pplacer [156]). Tree topology can be confirmed using a variety of other tree-building software (e.g., FastTree 2.1.7 [157], PhyML [118], RAxML [158], IQ-tree [159]) and methods (e.g., MrBayes for Bayesian tree-building [160]).

Information on virus abundance or activity can be inferred by mapping trimmed metagenomic or metatranscriptomic reads back to viral contigs normalized for between-sample comparisons (e.g., internal standards, library size, length, and reads per kilobase of transcript per million mapped reads [RPKM] values). However, there are some caveats to consider when examining environmental metatranscriptomes. Transcript abundance is not directly related to viral abundance for two reasons: First, biases are known to exist for highly transcriptionally active viruses, and second, single host organisms can support high viral loads. Moreover, virus metatranscriptomes can be contaminated with chimeras generated during assembly, remnant viral genes may be expressed from cells [161], and genomic duplications of marker genes could confound expression profiles. Some problems can be avoided with proper sampling and sequencing approaches mentioned previously, but others remain a significant obstacle for quantitative community analyses, though this has been resolved for bacteria-infecting viruses [147,162]. Until these confounding issues can be remedied and benchmarked for all viral types, they must be considered during the analysis of environmental data. A recent review by Nooij et al. provided a comprehensive description of workflows that have been produced for viromic analyses, including specific applications, classification biases, and open-source availability [153].

### 3.3. Other Downstream Applications of Omic Assemblies

Another enticing application of community sequence data is the potential to deduce biological interactions using co-occurrence or network analyses. This is a relatively new approach that was developed for microbiome communities but has the potential to identify novel virus-host pairs [163]. Two studies tracking the temporal dynamics of virus communities have been reported thus far [12,33]. From a metagenomics standpoint, these studies were striking because they generated putatively full-length Picornavirales and virophage genomes. Moreover, in the case of Moniruzzaman et al., 2017 [12] the viral genomes were generated from transcripts, indicating these virus genomes were actively expressed and were therefore, produced from infected cells. Beyond these exciting findings, each study used network analyses to link potential virus-host pairs. Clusters created from sequencing data collected over the course of a brown-tide bloom (*Aureococcus anophagefferens*) linked the brown alga to its known virus, AaV, demonstrating the ability to extract known relationships with this approach. Several other clusters were generated from the same study, including smaller networks of single virus-host pairs and expected associations between *Prasinophyceae* and *Phycodnaviridae.* Roux et al., 2017 [33] focused on using networks to link virophage with giant NCLDV hosts and found strong specific associations with *Mimiviridae* and their extended alga-infecting members to drastically expand the diversity of known virophage hosts.

Altogether, predictions stemming from the studies noted above demonstrate how network analyses can generate testable hypotheses for future studies of algal virus-host interactions. By deducing sequences of virus-host pairs, one can attempt to confirm probable virus-host interactions. For example, a variation of fluorescent in-situ hybridization, deemed phageFISH, could be used to label virus and host genes in infected cells [164]. Additionally, networks predicting viruses of cultured algae could be followed up with virus tagging experiments [165]. It might even be worthwhile to use more than one network building approach to look at ecosystem structures. Weiss et al. used real and mock in silico data to benchmark eight methods used for bacterial network analyses and found that some methods generate drastically different outputs [166]. This is explained, in part, by differing strengths for detecting particular biological relationships (e.g., mutualism and commensalism) across different network approaches. It was also suggested that *p*-values of 0.001 should be used for high-precision network detection and rare OTUs should be removed prior to network construction.

## 4. Conclusions

The opportunities for algal virus ecologists are at an all-time high. Bioinformatic tools are becoming more accessible to a wide variety of scientists through the creation of publicly available genomic databases and graphic interfaces that mediate interactions with traditional command-line software [167]. At the same time, researchers are increasing collaborations with one another by sharing methodologies in an interactive framework on *protocols.io* (e.g., Viral Ecology Research and Virtual Exchange network, or VERVE Net; https://www.protocols.io/groups/verve-net) and with cross-discipline collaborations fostered at research workshops funded by organizations like the *Gordon & Betty Moore Foundation* (GBMF) and the *Canadian Institute for Advanced Research* (CIFAR). The development of long-read sequencing methods, preemptively deemed “third-generation sequencing”, may address many of the issues with short-read assembly and viral quantification. DNA barcoding has been suggested as a cheap, reliable method to quickly track virus populations, and has recently been shown to recapitulate general viral community structures using sample volumes no bigger than a cup of water [168]. New virus isolates can be discovered from sequencing of single aquatic viruses sorted by flow cytometry, [169], as closely related, hyper diverse viruses are suggested to be difficult to assemble from metagenomes [170]. Even better, isolation and sequencing of infected single-cells may allow for the identification of new virus-host systems. Network analyses of community sequence data predict ecological structures that may lead to the discovery and isolation of several new algal-virus systems, bringing the scientific community “full-circle” to studying these systems in the lab. In light of that, this exciting frontier cannot be appreciated without recognition of the early work done by some of the first aquatic virologists in the field.

James L. Van Etten, for whom this special issue is in honor of, has spent the last forty years laying the foundation for aquatic virology. Not only did he open doors for other algal virus researchers to join the field, but he has set the standard for characterizing the biology and ecology of isolated algal virus systems. Along with the genomic, transcriptomic, and proteomic work done in the *Chlorella* virus system, the Van Etten lab has also shown that *Chlorella* viruses are biochemically novel in multiple ways. Virion proteins are glycosylated using a unique viral encoded machinery [171,172], and the viral genomes can be methylated by a range of DNA methyltransferases [173]. Many of these enzymes are paired with a restriction endonuclease that recognizes the same nucleotide sequence to comprise a viral restriction-modification system that recycles host DNA for virus replication [174]. *Chlorella* viruses have the smallest potassium ion channels that function to depolarize host membranes, concomitantly inhibiting secondary infection and host metabolite transporters [175]. Within seven minutes post-infection, transcriptional activity begins to shift away from the infected host towards producing viral transcripts [20,176]. Along with the extensive biological studies on this system, the Van Etten group has also established many important findings on their ecology. *Chlorella* viruses are ubiquitous in freshwaters across the globe, despite their hosts being sequestered as endosymbionts of *Paramecium bursaria* [6]. This inspired questions about their resistance to degradation as well as how viruses and hosts make contact with one another. Predatory activity on *Paramecium bursaria* catalyzes this contact by making the endosymbiotic algae available to *Chlorella* viruses in the environment [177,178]. Another group has shown that *Chlorella* viruses are more resistant to environmental degradation than other algal viruses, and can even overwinter under ice [179]. Collectively, these questions can be investigated in many types of algal viruses. Although dsDNA viruses certainly have the largest number of genes, even smaller DNA and RNA viruses must deal with many of the same selective pressures. Indeed, there are many lessons algal virus researchers can learn from the body of work produced by James L. Van Etten and his collaborators.

## Figures and Tables

**Table 1:** Algal viruses currently in culture collection.

Host Algae	Type	Size (kbp or knt)	Code	References
**Chlorophyceaea**				
*Tetraselmis* spp.	dsDNA	668	TetV	Schvarcz et al., 2018 [45]
*Tetraselmis striata*	dsDNA	31	Tsv-N1	Pagarete et al., 2015 [46]
**Trebouxiophyceae**				
*Chlorella variabilis* NC64A	dsDNA	287–369	PBCV-1	Jeanniard et al., 2013 [17]
*Chlorella variabilis* Syngen 2-3	dsDNA	327	OSy-NE5	Quispe et al., 2017 [18]
*Chlorella heliozoae* SAG 3.83	dsDNA	288–327	ATCV-1	Jeanniard et al., 2013 [17]
*Micratinium conductrix* Pbi	dsDNA	302–329	CVM	Jeanniard et al., 2013 [17]
**Mamiellophyceae**				
*Ostreococcus lucimarinus*	dsDNA	182–196	OlV1	Derelle et al., 2015 [47]
*Ostreococcus tauri*	dsDNA	184–192	OtV5	Weynberg et al., 2011 [48]
*Ostreococcus mediterraneus*	dsDNA	193	OmV1	Derelle et al., 2015 [47]
*Bathycoccus sp. RCC1105*	dsDNA	187–198	BpV	Moreau et al., 2010 [49]
*Micromonas pusilla* CCMP1545	dsDNA	186–195	MpV-02T	Martinez Martinez et al., 2015 [50]
*Micromonas pusilla* LAC38	dsDNA	173–205	MpV1	Finke et al., 2017 [51]
*Micromonas pusilla* LAC38	dsRNA	25.5	MpRV	Brussaard et al., 2004 [52]
*Micromonas polaris*	dsDNA	191–205	MpoV	Maat et al., 2017 [53]
**Pyramimonadales**				
*Pyramimonas orientalis*	dsDNA	560	PoV	Sandaa et al., 2001 [54]
**Rhodophyta**				
*Chondrus crispus*	dsRNA	6	CcV	Rousvoal et al., 2016 [55]
**Dinophyceaea**				
*Heterocapsa circularisquama*	dsDNA	356	HcDNAV	Ogata et al., 2009 [56]
*Heterocapsa circularisquama*	ssRNA	4.4	HcRNAV	Tomaru et al., 2004 [57]
*Heterocapsa pygmea*	dsDNA	ND	HpygDNAV	Kim et al., 2012 [58]
*Gymnodinium mikimotoi*	ND	ND	GM6/GM7	Onji et al., 2003 [59]
**Bacillariophyta**				
*Chaetoceros* cf. *gracilise*	ND	ND	CspNIV	Bettarel et al., 2005 [60]
*Chaetoceros salsugineum*	ssDNA	6	CsalDNAV*	Nagasaki et al., 2005 [61]
*Chaetoceros setoensis*	ssDNA	5.8	CsetDNAV*	Tomaru et al., 2013 [62]
*Chaetoceros socialis* f. *radians*	ssRNA	9.4	CsfrRNAV	Tomaru et al., 2009b [63]
*Chaetoceros lorenzianus*	ssDNA	5.8	ClorDNAV*	Tomaru et al., 2011 [64]
*Chaetoceros tenuissimus*	ssDNA	5.6	CtenDNAV-I*	Tomaru et al., 2011 [65]
*Chaetoceros tenuissimus*	ssDNA	5.6	CtenDNAV-II*	Kimura and Tomaru 2015 [66]
*Chaetoceros tenuissimus*	ssRNA	9.4	CtenRNAV	Shirai et al., 2008 [67]
*Chaetoceros tenuissimus, Chaetoceros* spp.	ssRNA	9.6	CtenRNAV-II	Kimura and Tomaru 2015 [66]
*Chaetoceros* spp. SS628-11	ssDNA	5.5	Csp07DNAV*	Kimura et al., 2013 [68]
*Chaetoceros* spp. TG07-C28	ssDNA	ND	Csp05DNAV	Toyoda et al., 2012 [69]
*Chaetoceros debilis*	ssDNA	ND	CdebDNAV	Tomaru et al., 2008 [70]
*Chaetoceros* sp. SS08-C03	ssRNA	9.4	Csp03RNAV	Tomaru et al., 2013 [71]
*Chaetoceros* cf. *wighamii*	ssDNA	7-8	CwNIV	Eissler et al., 2009 [72]
*Asterionellopsis glacialis*	ssRNA	9.5	AglaRNAV	Tomaru et al., 2012 [73]
*Thalassionema nitzschioides*	ssDNA	5.5	TnitDNAV	Tomaru et al., 2012 [73]
*Rhizosolenia setigera*	ssRNA	11.2	RsetRNAV	Nagasaki et al., 2004 [74]
*Skeletonema costatum*	ND	ND	ScosV	Kim et al., 2015 [75]
*Stephanopyxis palmeriana*	ND	ND	SpalV	Kim et al., 2015 [76]
**Pelagophyceae**				
*Aureococcus anophagefferens*	dsDNA	370	AaV	Moniruzzaman et al., 2014 [23]
**Phaeophyceae**				
*Ectocarpus fasciculatus*	dsDNA	340	EfasV	Kapp et al., 1997 [77]
*Ectocarpus siliculosus*	dsDNA	320	EsV	Kapp et al., 1997 [77]
*Feldmannia irregularis*	dsDNA	180	FirrV	Kapp et al., 1997 [77]
*Feldmannia simplex*	dsDNA	220	FlexV	Kapp et al., 1997 [77]
*Feldmannia species*	dsDNA	170	FsV	Henry and Meints 1992 [78]
*Hincksia hinckiae*	dsDNA	240	HincV	Kapp et al., 1997 [77]
*Myriotrichia clavaeformis*	dsDNA	320	MclaV	Kapp et al., 1997 [77]
*Pilayella littoralis*	dsDNA	280	PlitV	Maier et al., 1998 [79]
**Raphidophyceae**				
*Heterosigma akashiwo*	dsDNA	ND	HaV	Nagasaki et al., 1997 [80]
*Heterosigma akashiwo*	dsDNA	180	O1s1	Lawrence et al., 2006 [81]
*Heterosigma akashiwo*	ssRNA	9.1	HaRNAV	Tai et al., 2003 [82]
*Heterosigma akashiwo*	ND	ND	HaNIV	Lawrence et al., 2001 [83]
**Haptophyta**				
*Emiliania huxleyi*	dsDNA	415	EhV	Castberg et al., 2002 [84]
*Phaeocystis globosa*	dsDNA	466	PgV-16T (Group I)	Baudoux et al., 2005 [85]
*Phaeocystis globosa*	dsDNA	177	PgV-03T (Group II)	Baudoux et al., 2005 [85]
*Phaeocystis globosa*	dsDNA	176	PgV-102P	Wilson et al., 2006 [86]
*Phaeocystis pouchetii*	dsDNA	485	PpV	Jacobsen et al., 1996 [87]
*Chrysochromulina brevifilum*, *Chrysochromulina strobilus*	dsDNA	ND	CbV	Suttle and Chan 1995 [88]
*Chrysochromulina ericina*	dsDNA	510	CeV	Sandaa et al., 2001 [54]
*Chrysochromulina parva*	dsDNA	485	CpV	Mirza et al., 2015 [89]
*Haptolina ericina, Prymnesium kappa*	dsDNA	530	HeV-RF02	Johannessen et al., 2015 [90]
*Prymnesium kappa, Haptolina ericina*	dsDNA	ND	PkV-RF01	Johannessen et al., 2015 [90]
*Prymnesium kappa*	dsDNA	507	PkV-RF02	Johannessen et al., 2015 [90]
*Prymnesium parvum*	dsDNA	ND	PpDNAV	Wagstaff et al., 2017 [91]
**Cryptophyta**				
*Teleaulax amphioxeia*	ND	ND	TampV	Nagasaki et al., 2009 [92]

Table 1. Summary of all reported eukaryotic algal viruses that have been isolated. A range of genome sizes (kbp or knt) represents multiple virus strains associated with the same host species, and in this case, only the type virus is reported under the code column. Asteriks denote original names for some of the diatom ssDNA viruses, which have since been renamed and placed into genera of the family *Bacilladnaviridae* (*Chaetoceros setoensis* DNA virus = *Diatodnavirus*; *Chaetoceros salsugineum* DNA virus 1 = *Chaetoceros protobacilladnavirus* 1; *Chaetoceros* sp. DNA virus 7 = *Chaetoceros protobacilladnavirus* 2; *Chaetoceros lorenzianus* DNA virus = *Chaetoceros protobacilladnavirus* 3; *Chaetoceros tenuissimus* DNA viruses type I and II = *Chaetoceros protobacilladnavirus* 4). ND = Not detected or reported.

## Supplementary Materials

The following are available online at http://www.mdpi.com/1999-4915/10/9/487/s1, Supplemental Tables S1 and S2 contain the full information for amino acid sequences used to construct RdRP and polB phylogenetic trees, respectively.
