# Supplementary material for: Viruses of Eukaryotic Algae: Diversity, Methods for Detection, and Future Directions
Source: Viruses. 2018 Sep 11;10(9):487. doi: 10.3390/v10090487 (PMC6165237; doi:10.3390/v10090487)
Supplement: Supplementary file 1 [file viruses-10-00487-s001.zip › 5-viruses-347578-supplymentary material/4-viruses-347578-supplymentary.docx]

Supplymentary Material

**Supplemental Table S1:** RdRP amino acid sequences used for phylogeny of ssRNA viruses.

| **ssRNA Virus Name** | **Accession** | **Trimmed Sequence for ‘CDD 01699’ RdRp domain** |
| --- | --- | --- |
| *Foxtail Mosaic Virus* | ABW25054.1 | fnpelwwacadevqktylskpihalkngilrqspdfdwnklqiflksqwvkkidkigkidvnagqtiaafyqptvmlfgtmarymrrirdtyqpgeilinceknqkhiskwvesnwnhrlpaytndftaydqsqdgamlqfevlkalhhdiphevveayvalklnskmflgtlaimrltgegptfdantecniaytharfeipkdvaqmyagddcalncrpverqsflplvekftlkskpkvfeqkvgswpefcgnlitprgylkdpmklqhclqlaqrkkpsepgslkdvaeny |
| *Indian Citrus Ringspot Virus* | NP_203553.1 | tylskplaalanaaqrqdpdfdsnkiqlflksqwvkkveklgclkikpgqtiasfmqqtvmlygtmarymrririslcpshimincetnptqisswvrenwdfsgqshendfeafdqsqdanmlqfelikakfhsipeeiiagykhlkchahiflgtiaimrlsgegptfdantecsiaynhtryfvpkgcaqlyagdds |
| *Heterocapsa circularisquama Virus* | YP_386495.1 | fvkdephtadkarremwrliwnvslvdsicqayfnrelnlqqnrdyqgghpvphtcgmghhdegikrlgeaieaafpdgivcssdasgwdmsvsrdglifdglvrairtqdpsatayhnigtsvcilldkfvqsahmictgtslwsvdvygitasglpdtttqnsfvrgmgaklagcfkaltagddllcdnrlrlpvltehgtitkgdvttanwrkgqpvgftshslvrgpdgqw |
| *Turkey Astrovirus* | NP_853540.1 | wwcflktevlklakieqddirmilctdpvftrigaafeqhqnslmkletenhhaqvgwspffggihrratrlygehryyveldwtrfdgtippelfrriklmrfflldpkyktpenrdrynwyvenlidkvvllptgevckiyggnpsgqfsttvdnnfvnvwltvfelaylfykehnrlpticeikkhtdwicygddrllavdkrfinsydtaaviamykdvfgmwvkpdnikvfpslegvsfcgmvwtkrkgqyvgkpnvdkilstls |
| *Bat Coronavirus* | ATO98216.1 | fvvevvdkyfdcydggcinanqvivnnldksagfpfnkwgkarlyydsmsyedqdalfaytkrnviptitqmnlkyaisaknrartvagvsicstmtnrqfhqkllksiaatrgatvvigtskfyggwhnmlktvysdvetpnlmgwdypkcdrampnmlrimaslvlarkhstccnlshrfyrlanecaqvlsemvmcggslyvkpggtssgdattayansvfnicqavtanvnallstdgnkiadkyvrnlqhrlyeclyrnrdvdhefv |
| *Turkey Coronavirus* | CBL62866.1 | aaslvlarkhtncctwseriyrlynecaqvlsetvlatggiyvkpggtssgdattayansvfniiqatsanvarllsvitrdivyddikslqyelyqqvyrrvnfdpafvekfysylcknfslmilsddgvvcynntlakqglvadisgfreilyyqnnvymadskcwvepdlekgphefcsqhtmlvevdgelkylpypdpsri |
| *Phytophthora infestans RNA Virus 1* | YP_003193667.1 | dhrwyttgraklvkmgkpdkarlvlysgfsysllgfvysqvwtgfmnrqcrgwsavgmswmnggaakvasffedcfgiavsgfeymtldvaewdssvcrellhackrfhmrvlertlspenarykeyfgriydemieakvvlpgghsfrlhhgmksgwimtandntlmhefvvrtlqkigqipdmkrqlygddnlsrkpigmskqllvdgygmfgfrlshihvsrrlsevdflskfiifkdgfyfpwreqtetharl |
| *Barley Yellow Mosaic Virus* | BAG70353.1 | igqmnkglidkhvivgenddvydfmrehptftwlkdfmneyapsvlsysayykdlckynrakhvltynpeelhyatkglikmledagltqgsvrtpqqvisdiqwntsagpsyqgkkrdlcahlsddevlhlaevcrqqflegkstgvwngslkaelrtiekveaektrvftaspitslfamkfyvddfnkkfyatnlkaphtvginkfgrgwerlhdklnrpgwlhgsgdgsrfdssidpfffdvvktirkhflpsehhkaidliydeilntticlangmvikknvgnnsgqpstvvdntlvlmtaflyayihktgdrelallnerfifvcngddnkfaispqfdeefghdfspelvelgltyefdditsdicenpymsltmvktpfgvgfslpveriiaimqwskkggvlhsylagisaiyesfntpklfksiyayllwlteeheaeila |
| *Bovine Diarrhea Virus* | AAF82566.1 | hlveqlvrdlkagrkikyyetaipknekrdvsddwqagdlvvekrprviqypeaktrlaitkvmynwvkqqpvvipgyegktplfnifdkvrkewdsfnepvavsfdtkawdtqvtskdlqligeiqkyyykkewhkfidtitdhmtevpvitadgevyirngqrgsgqpdtsagnsmlnvltmmyafcestgvpyksfnrvarihvcgddgflitekglglkfankgmqilheagkpqkitegekmkvayrfediefcshtpvpvrwsdntsshmagrdtavilskma |
| *Tomato Ringspot Virus* | ABG23688.1 | eqartevplligmdvpkderlkpskvlekpktrtfvvlpmhynlllrkyvgilcssmqvnrhrlacavgtnpysrdwtdiyqrlaeknsvalncdysrfdgllnyqayvhivnfinrlyndehstvrgnllmamygrwsvcgqrvyevragmpsgcaltviinslfnelligyvyrvtvprslvnnfkqevclivygddnlisikpdtmkyfngeqikstlakyrititdgsdknspvlrakplkqldflkrgfrvesdgrvlapldlqaiysslyyi |
| *Heterosigma akashiwo RNA Virus* | NP_944776.1 | demiaclerglpvpclfvatkkdealrigkvprtfyaasmnvimavrkyfcpvlqalkanpihaeiaigtnafgkdwadiyshlashstetviagdyssfdmshnadavrcamqvlldlidesslysdvdklaartlveslgqsflafdgtwmqvigwvmsgvpltaelsstlnqiymrvvwkvvtqrpisdfrshvalivygddnnaavrdeprynfqsvavtmgkfrmtytntdkndemhiyqrledaeflkrlwvpgplkvyaplswdsinkrivwt |
| *Acute Bee Paralysis Virus* | NP_066241.1 | vrtpvmwvdtlkderrpiekvdqlktrvfsngpmdfsitfrmyylgfiahlmenritnevsigtnvysqdwnktvrklktmgpkviagdfstfdgslnvcimekfadlanefyddgsenalirhvllmdvynsthicgdsvymmthsqpsgnpattplncfinsmglrmvfelcskkysa  lngtkcyvmkdfskhvsivsygddnvinfsdevsewfnmetiteafeklgftytdelkgkngevpkwrtiedvqylkrkfrydskrkvweaplcmdtilempnwc |
| *Cricket Paralysis Virus* | NP_647481.1 | lrrdveelidncakgiikdvvfvdtlkderrpiekvdagktrvfsagpqhfvvafrkyflpfaaylmnnridneiavgtnvystdweriakrlkkhgnkviagdfgnfdgslvaqffgqscgksfypwfktfndvntedgkrnlmiciglwthivhsvhsygdnvymwthsqpsgnpftviinclynsmimrivwillarklapemqsmkkfrenvsmisygddnclnisdrvvewfnqitiseqmkeikheytdegktgdmvkfpslseihflkkrfvfshqlqrtvaplqkdviyemlnwt |
| *Triatoma Virus* | NP_620562.1 | leekvkevidlakqgvryshvfvdalkderkprekahktrafsgcpleylavckmyfqgivsvltkcknethisvgtnvyskdwdfmarylksksdgfvagdfegfdssqlvpilreignvfngiarqfpdwkpeddevrlvllqslwhsihinggdvvmwghalpsghyltapynslyatmlfsmafvilsrrngtrmgpsmlaskffkefgfvaygddhicavpkryqsffnqmtlekvflelgigyttedkreidvpirsldeiaylkrsfvldeerqqwiapltldtvletpswi |
| *Black Queen Cell Virus* | NP_620564.1 | lrekvqecieaarqgkildhyfidtlkderkpkhkahksrmfsngpidylvwskmyfnpivavlselknvdhisvgsnvystdwdviarylkskshhmvagdfegfdaseqsdilyaagevlqelskkifnstedemlqqraiihslvnslhinengivlqwckslpsghyltaiinsvfvnlvmclvfmeanqkysfttassffrecgivaygddhvvsvpekylsvfnqqtlpvlmskfgmfytietkddteidflsrrledvsylkrnfvydesrqryiaplsldvvlempmwt |
| *Varroa Destructor Virus* | AGO86045.1 | phtiftdclkdtclpvekcripgktrifsispvqftipfrqyyldfmasyraarlnaehgigidvnslewtnlatslskygthivtgdyknfgpgldsdvaasafeiiidwvlnyteeddkdemkrvmwtmaqeilapshlcrdlvyrvpcgipsgspitdilntisncllirlawqgitdlplsefsrhvvlvcygddlimnvsdemidkfnavtigdffsrykmeftdqdksgntvrwrtlqtatflkhgflkhptrpvflanldkvsiegttnwt |
| *Antheraea pernyi iflavirus* | YP_009002581.1 | fvdclkdtcidikkcsipgktrifsispvqytiafkqyfgdfiasyqearlsaehgiginvdslewsqvanyittygdniiagdyknfgpslmlkcvekafdiimnwyerydndeerqlirrvllseilhaqhlclnvvygvpcgipsgspittplnslvnslylrcgwksitnqnfstmhenvriltygddvcinvsdmykdiyntetlslffkeynivftdidksdviikyrnlnnvsflkrsfilhpnskfiflapielqsirkcvnwi |
| *Sacbrood Virus* | AAL79021.1 | krkselrrqgvqpitpfidtlkderklpekvrkyggtrvfcnppidyivsmrqyymhfvaafmeqrfklmhavginvqstewtllaskllakgnnictidysnfgpgfnaqiakaamelmvrwtmehvegvneieaytllheclnsvhlvsntlyqqkcgspsgapitvvintlvnilyifvawetlvgskergqtwesfkqnvelfcygddlimsvtdkykdifnaltisqflaqygivatdankgeeveayttllnstflkhgfrphevyphlwqsalawssindttqwi |
| *Chaetoceros tenuissimus RNA Virus I* | BAG30951.1 | eylagrraypvfkactkdeptkltkskvrvfqaapakvqynmrkyylsiakflsdhpllsecavginsqgpqwheldthiskfgrrgrtvagdfkaydqhmsarmvmlafkimikiaeksgnyssddikimkglmtdvafpvmmvngdlvqlfgsnpsgqnltvyinsivnalyhrcaffeiypdfeglfhevvalttygddvkfsvskmfdkynhtniqkifakrgieytmaekdaqsvpyldhvdadflkrqsewraeyswkgskgmwiaklsedsifkslhc |
| *Rhizosolenia setigera Virus* | YP_006732323.1 | larkarlawlddkrsydifktctkdeptkitkdkvrcfqaspvslqfnirkyfltlchflssaslvsecavginsqgrgwhelnqhmikygknrivagdfkaydqhmsarmtlmaakvfeyiaglagyteeelkimrgastevsypvmslngeliqlygsnpsgqnltvytnsivnslyhrcafrkiypnfsgryrdavalmtygddvkmsvspkfpdynhtriqsefnkqgieytmaekeaasvpfiqhedadflkrksrwepsysytegdgtinkgmwlamldeesifkslha |
| *Marine RNA Virus JP-A* | YP_001429581.1 | ikacvkdzatkigkekvrvfeaadwafqlivrkyflpiarmlslfpltsecavgvnaqgpewdqlarhmkkfgadrifagdyskydlrmpaqlilaafkclidiaktcgqysvddikimqgvateiayscvsyngdliihcgsnpsgqnltvyincivnslllrcayyhmypaaegnpepfrhncavmtygddvkgsvrqecdwynhityaqflaerdmvftmpdkestptpymndldadflkrhnlynpetglihgvldqnsifkslhsv |
| *Chaetoceros tenuissimus RNA Virus II* | BAP99822.1 | aifkacvkdeptplskdkvrvfqaaswafqllvrkyflplarlmslfplqsecavginahgpewdeyakfmkqhgddrilagdyskfdlrmpaqmlmatykvfcnvcekcgtyseddltimrgiateisysvvayngdliihngshpsgnnmtvygncgdnclnfrcgfaynglkngytlktlpkfksvcalgtygddakgsvkkgfdwfnhitfadymakndivftmpdkestptkymkdtdadflkrknvfneetglihgaldedsifkslhtv |
| *Asterionellopsis glacialis Virus* | YP_009047193.1 | lweevkrledilasgkrintvfkgslkdeptkmtkdkvrvfaacnfatillvrkyylslaalvqrnqklfecavgvvqqspewtdifkhigkygwergiagdyakfdarmsarfmlaafkiliqvaeksgnysdrdlvimrglateityptydyfgtlvqffgsnpsghpltviinslvnslymryayytiakeeswwrtpafrevvslmtygddnimsvkkgypginhtriaevfasmgikytmadkdaesvpyidlstasflkhfavwddelklyrcpceegsiakmlha |
| *Marine RNA Virus SF-1* | AFM44930.2 | mnkervhpvycghlkdepvtfekaisgktrvftasslahtlvvrmyllpiivhlqnnrftyelgpgtivqslewqkiheyitefgqdrivagdyskfdkrmpanvilaafeiienicaragydegdlnvvrgiaydtayplvdfhgdliefygsnpsghpltvivnglanslymrycyivlrpigaasrkfrenvklmtygddnimgvaetcpwfnhtaiqttlqnvdigytmadkdadsvpyihisqanflkrtwrwdedigalvapldrsslnkml |
| *Marine RNA Virus JP-B* | YP_001429583.1 | ahlkdepvsfkkakvgktrvftgatmdwylivrkyllsfarllqnerfafeaapgtiaqslewhelydyvvkngedrviagdykafdkrmspkeilaafdiiiyfcqlsgnyseediqiirciaedtafalvdyngdlvqlygsnpsgnpltvilnsivnslrmryvyfmlnpehtvttfkdnvnlmtygddnimsvsrecdwfnhtsisetfatlnivytmadkesasvpfinikdasflkrtwrmddqlgcyvapleeesiekslm |
| *Beihai Picorna-like Virus 63* | YP_009333515.1 | lkdeprtfeksqagktrifamssypmtlvnrmylmpfyalmceyreifgtrvginmhsneadkmyndlvsfsknimegdyggydtsmpvdigliansivfnvlkqlgynefalkivkgilcdnlyptmamegniiqvpgfqpsgkyataednslrglilmyyafaemctplgkgsihnktidynindffklikpetygddmlasvkdsislfynnityssfvsdvygmefttadkhghtskfvdctkisflkrsfvynpllgrivavldrdsfvkslsyi |
| *Aurantiochytrium ssRNA Virus 01* | YP_392465.1 | lkdepraykkivdrktrvfcmspyestlvnrmylmpfytlmvehgdifrtaiginmhsqdvgdlvtrmtdfsdqfmegdyggydtsmpydiglaantvvyqvcqdlgydshalqmvrgilsdnlyptvvmrgdvfaapalqpsgkyataednslrglilmvyaisectdigdqctgrarttqfqpedfftqvlpviygddmlagvkpvaqqfynnntyqtfckevygleftnaqktaemanflewddtsflkrsfvfredlqvwvaqlelasimksicyy |
| *Wenzhou Picorna-like Virus 24* | YP_009336770.1 | vtqcalkdevrsisknklaktrvfcvgdfsmlivsrmylgplftlmqqssesfgcaigintheqadklarfltgfsdrfmagdyseydtrmppiitlsantvlfmlarkygyddkqliivrgilsdslfplylmdgllmssagstpsgeygtaernslkglillmlgynilrptdipletfednvlpvtygddlvagikshicdwfnnvaygaytislgmnytdpkkrevmvpycsiedvtflkrsfvynpltdkwnmplewdsigksmsy |

**Supplemental Table S2:** polB amino acid sequences used for phylogeny of dsDNA viruses.

| **ssRNA Virus Name** | **Accession** | | **Sequence** |
| --- | --- | --- | --- |
| *Aureococcus anophagefferens Virus* | YP_009052217 | masfqiydwfpqdelinededsdnevsekqnyvihlfgvnkdgknvhckvkdftpyfyielpenwkkswtslfmeklkdslpksiqdefvcderqinravklkykfrnyqwktpkkfmqlvfksesaarflyyklkkpfsfttarllkhnfpiyeknvlpvlrfihlrkiqpsswirlenykqidsklaeskfeinyevnwrnvfpdevdgsasikiasfdiecdsshgdfpvakkdyqklasniyeeylrllrnnkkvnptlvkqwincafrditnekyddsmkssiqtiylknkthfktiteeeieklsmkilgilikkmkgketiesinnilndnlpsvkgdkviqigtviqnysnpsdikrhivtldgcsdltgievetaktvdelifkwvkmmkreqpnivtgynifgfdikflwecaeefgclkylrkigalknqectmetkelysaalghnflyyfnspgivyidlmkvvqkdhnlssyklddvstqfinsgitqfehfednvkvytkstfslkvgdyiaifkqtiignefvgekrkilemtenesfilegegndfpenpksfcwsvgkdnvspqdifdmqrgsdedrsvvakycvqdcelclnimqkleivtnnlgmsnvclvpfsylfmrgqmiktlslvssecqkenylipelprpkedtkdsyegaevleptpaiflnepvsvldysslypssmigsnishdsiikepkyqgetgaklleemgvkfedvyydnyinrlvgktwkktiveeepvvscrfiqptlledgsidnnsrgilprilmkllkarkdtrnliktekdafrrsvldglqlaykvtanslyggvgaevsslyykdiaasttaigrrhlhlakdyvkehfpkadivygdsvtgytpitikykeqifiekienvakifgedkwqkcidpgkqekeacelnecftwtskgwtklhrvirhilvkekkiirvlthtccvdvtddhslilksgeeispkdlkigdellqrdiefdeiieysndeyvkkqikylkenmksenesilslneidykgyvydlttdnhefqagigniivhntdsifvnfkpnasgkegiqesidrsveveegiqkllayphkleyektfspfillrkkgyignkyefdldkftqtsmgvvtkrrdnagivkyvydgiikrimndrdidsairfllttiddildgkfplqyfiitkrlnavyanpaaivhkvladriadrdpgdkpqsndripyvyiqtkeepklqgdrvehpnyiiehklkvdylfyitnqvskpccqvmglalehlrkygyrlpanhfelirekleregkkskseirerimeerakeaynvlfksrvkveegkkygqtsitsffkkk | |
| *Acanthocystis turfacea Chlorella virus 1* | YP_001427279 | mdiypvdwrsgdengqfkitmfgktvdgksscvrirftpsfllempdswsaprqrlfitetvmkygaikdmclpvqrkslwgfdngksrnmaqfafptmeamrkakyglkgnhqvyessvdpiirlfhlrklspsgwvrvsqsypamtrvsrcdveiecnftqvgpsdvvttpplviaswdietysrerkfplasnpddyvtqiatsfqrygeaepfkrvvfclndtadvqgveivscsqeadvinawmdaitkektdvligynvfqydwkyvygraqmlvddataeetvfpeklgylledggkvverelasnafgqnffyyldtpgviqldllqwfrknrnlesyslnnvsklylgdqkddlpamkifekfegdaedraviakyaaqdtllplkllsklaifedvaemanavkipmdwvgfrgqqvrafsclfgkaremgyaipdnkawpaegkfegatvlepkkgayfepiaaldfaslypsiirahnmspetivmepkfanvpgveyyeiqtgigkfrysqksqgvvpallddlakfrknakklmaqahkegddfkealydasqrsykvvmnsvygflgaskgfipcvpiaasvtatgrnmidvaarralellpgsvvvygdtdsimvkmklpegkdqnnindhfevakwlageitkeykapndlefekiyypyilyskkryaaikfedpnekgkvdvkglalvrrdfspitrdilkesldtilfakdtptavkdtrekirkvldneypmekfvmsktlktgyknemqphlivankilertgvpvpsgarvpfvyvedddkidlkqsmraedptfaqnnglvvdrlfyidhqllkplvslfdplvddpekelfghedvvekiemlknvhnkqlkvtkrvkknasnrqheitsffktkgk | |
| *Bathycoccus sp. Virus* | AHA82625 | | Vldaqkgayytpitaldfeglypsimmahnlcyssmvmdskyenipgityetfgfykfaqdvpsllpsillelkqfrkqakkdmaqssgalkemyngkqlaykvsmnsvygftgaskgmlpcvqiastvtlkgrsmidetkayveknfpgakvrygdtdsvmvefdvgnrtgkeaieysweigeraaeectklfkapnnlele |
| *Chrysochromulina brevifilum virus PW1* |  | | egatvldatpgahfepiagldfaslyasimiahnydyativedpefdnlpgieyfdmdweeddidsdgneikrpvsvrfvqnrtgimpkildrlwkerkairkqmktlspddplyavyngvqlaikvsmnsiygftgarygrlpikkiaaavtacgrgmiahskkcaeewyecevvygdtdsiyvkfksdlkgrdhmnyvfkvapecadrisatfkkpidlefekvmypfilyskkryar |
| *Chrysochromulina ericina virus* | YP_009173620 | | msqwqfklfefdireeletdknefipgrdtkrfiiqmygidengktacifvkgfnpffyvkvldewdnskvtefvafvrkemgayfgdslvsakivyrhkfyefdnkklykfvqlkftsigafnkcknlwynetkygedrklkenglefldtkttlyeaqvppllrlfhirqikpsgwvalknghysqnrkqlttcdyeftvnykniypinderlekicvpfkilsldieassshgdfplarknylklatnivdylinnnietcdkdllsnliktgfsyrinkdiekiylkksiteeeldelidnlvdikpgkkenyidikdedensdssdiendgiveefttkkrqkvtgtknkdsnileiindltcnrntrilelakcfgqhnpnrdnkwegkfpelegdqvtiigstirrngedkpylqhaivvndcnsidnvviesykterdallawtnfvqrenpdiiigynhhgwdegfmydrsielncmtqfsklsrfknekcikeifqgknkpkkitieesstklasgqfdlryfkmsgrlqidflnlfrreeqlpsykldyvaghfigdnikkieydencsilysknltglskndyiviqeigystdqyangkkfkvldivddkiilndkitpdtnkilrwclgkddvgpqdifrltnegpegraivtkycikdcdlvqdlmrkndtmtsydemsnlcwvpksflvtrgqgikltsyvaqkcrekntlmpvidkgidgegyegaivlepkcnlylkkpvacvdysslypssiisenishdskvwtkeydlddnlinetgekdengkfiydelpeydyiditydtfkwirktpkaaatkvksgyktcryvqfqnnekailpsildellgarkstkrqmknetdpfmqnvldkrqlsikitanslygqagaktstfydkdiaasttatgrklliyakelieacydntieettnygevkcygeyiygdsvasytpiyvrynksiidicsveelaekygngwhlespkeycelnnieswtengwtechrvirhrlapykkmvrilthtglvdvtddhslvkntgeeispkdvsigtkllhctmsenesniesdisidearimgfffgdgscgiydcpsghkaswalnnsnkeliekyynlcksvypefewkvydtlnssgvykicfnkksgskskiqfiekyrsmlynkkskiipseiingsielrksfweglydadgdkdkngytridqksqisaayicwlansigyktslnirddktdiyritatknkqrrdgdkikkivniqnsaniqnsaniqnsvniqnsvniqnskdnqdyvydlttenhhfaagignmivhntdsvfftfnlkdldskeivgkkaleitielakkagwlatmflksphdleyektflpfcllskkryvgilyeedpnkgkrkemglvlkrrdncaqvkdvyggaidilmkdqvvhkavdfvkhslqdvidekisqqkliitkslrsyyknpkqiahnvlairigerdpgnkpkpgdrmefiyiknsdkkalqgdrietplfinennieidygfyitnqimkplqqlfalvledmedfvtkrgismkswkaeidklhekwtepdkfskkyeelrckevksilfdpyikllk |
| *Chrysochromulina parva Virus BQ1* | ALH45661 | | Egatvldatpgahfepiagldfaslypsvmiahnfdystiveseefdnlenityetikwdegevkfaqnykgimpkilerlwkerkairkqmkdlspedsmyavlngvqlaikvsmnsiygftgakygrlpnkliaasvtacgrqmiahskkcaeewyncevvygdtdsiyvkfksdfkgqehmdyvfrvapecanrisatfknpielefekvmypfilytkkrya |
| *Dishui Lake Phycodnavirus 1* | YP_009465906 | | Mvtfqviawddrdeddkhlisiygktedgksvcvttpytpyffvkfpsdwstsdahvfiqnlelkckgalvghefvdrkdmwgfqngelskfvrldfptlkarrlvdwkirdqfpkveafeanldpvlrfmhetniqatgwvraergtnpsfvahvdvdlwvddwrnlesverddvapfviasvdieaysqthkfpnaqiredacfqigvtlchigtdtpydeaifcygqtdpvdgvrtesftteagmlaafrdyiheknvdvvtgwnifgfdldylytralmtncqkffnlgrrrgfsskvvekklsssalgdnvlkllpmpgrfvydmfqevkknykldsyslnnvslvylndskidmparemfarferqnpkemsevaeycvkdtvlphrickrlcldvnllemakacwvplsylcergqqikvfsqvckkarelgflvktirskddpgsyvgatvldaqkgayyknpitaldfaslypsimmahnicystlvmdpqydnipgveydefhvagvtlryaqkvpsilpsilsdlkqfrkaakkqmanaegfmkqvfdgkqlamkismnsvygatgtsvgilpcvfkgcmalaatvttkgrsmieetknyveanfpgavvrygdtdsvmvefdcqgrtgmdaieyswklgelassgatklfrapndlelekiyhpfllyskkryaakmyemgksgnvefkkvdikglslvrrdttkhcrsvcrelldvilnssdpqpaidlararaislltgevpnselilsqtlsesykvkgepvsvtdelaslrinqahvavmrkmrerrpgsepqtgdrvpylivrtddpkakafeksedpayveqnklpvdyfhyfenkfstpvsdlleplvegdakreifgeirgqhrpkttrerkkkesdptereknaistlfknyaasmnk |
| *Ectocarpus siliculosus virus 1* | NP_077578 | | Melylhdirdnsgsfqnptmqlfameedgtnvfvsvknfktylyvgfdldisedsvrsnylekfkqekwernvykmsvvkrkrligfsngdlfpyilmeftgtisfyivrkhlhelcgerdpgpntfvdlnkypgmcvyesksvdsilkffhasgvrpssyfrmenyvrvadkarkthcakefivdfvnvrpvgeevvdrkpppmticsydletsglntnedyifqasmifsrlgdpcpdsegsatghavdsytdgvvicvgdtesvdgtpllivenelqlldkfreilvergcnilcgyntfkfdsaflykraerygfdgfkklsfikdlacdlevktlqsaalgknelkqiiipgrveidlfmvmrrsqklssyklnavcdkffggkkddvtyadilqactskdpkklgviakycyqdsglvlklldkikevydatemaklctvpltyivgrgqqikcmslilnrihgeyvcnyaaakkkmaadgkqvlnegykgasvidakkgfyekdpivtmdfaslypsimrlkqlcyttivrdvkyrgiegvnyedhqisdgvsvtfahrpgsrsilceleemlgeerkatkklmksekdpfayslldskqkaqkvtmnsiygftgtvnngmlplveiaaavtstgrdmikrtkeyaekehgcnviygdtdsvmvifpehrnienlgdkmrycfdmgtkvskeisemfghpillefeniyfkyllvskkryaglswetvegpptmtmkglvtvrrdnapfvgrcaseaihmlmdvdvtdgrgavkkhltetllrlergqisiedltirkelkqwvyktpsphatlalkilertkeqavfrefikpayetiggyddsllssvwtkmtnlksylsvrakreiamsdmvesirgdttspfkaeayavvalrqlyddvhsvlvgesfarvvglvmagigdvhklgerymafvrynivdwdpptlgeripyvittgkgdissraedprmvnvgrcrpdflyyidhqlrnpmvdllqhviespsslfvesqrrmsnlnhgrkeittffkkrkvteg |
| *Emiliania huxleyi virus 86* | AA_158859 | | egatvldaksgfytdpvatldfaslypsimlahnlcystfvekgtprvdgveyethkispteeytfalnvpgvlthmlkyllgarkkakkqmaaaktpeekaiynarqlalkiscnsiygfcgaeklgkyplgaiakcttfngrkminktsemaielfkpwiaeiiygdtd |
| *Emiliania huxleyi virus 201* | AET97918 | | msilgkyglraqppneyvstdiniqlmaievedlpiestldeeynesviasdqfistlppenaheyvkrkavaylfgaeehtgatvcvkvdkfrpvlyyhtndsvstlknkideslnlkgdidakvikrkrtygfhpdkddstsheqirivevsfpsvskmkaacyrsgdkeppetrlpkpweqgvdpgsmfmerngltpcgwfkmkacktitkhkishctmefevsnpkniqpvdldkiapiliasydfemysetrgfpmadrqgdhiaiigvafwrlgtpvedtktvllclnecapvegsyvesynteaelynafrdlitvhsdadvctgynifgfdneyittrakmckasrfayngrlitvktesqakelessalgqnrmfpiewkgrcnfdlfnfiksnhklslyglgpvsqhfigetkvdlpyqemfdcvrpgatpeevaraaayckgdvllpirlmkalqvmpgmiemsrvtfttinqlvfrgqsikvmqqitryshqlghvvnpvprpintsgyegaividaksgfytdpvatldfaslypsimlahnlcystfvekgtprvdgveyethkispteeytfalnvpgvlthmlkyllgarkkakkqmaaaktpeekaiynarqlalkiscnsiygfcgaeklgkyplgaiakcttfngrkminktsemaielfkpwiaeiiygdtdsvfvrvkdkngkeltpsevfkvgedvaqkisdsfredielemekvysgfllitkkryfggmhepnkagdvvfskvdakgvelvrrdncpllknlykkivdflvfdkdplkalasvketlervvndevpyeeyiitkelrkeesyanpkqeqlmlakkisartnggvtpqpgdripfvirydkhakhicdraedidylrennipldrlyyitnkitkpiltifqafkehihdvqrtiqsamsrvqlqldkqptitsffnklpplpvinmdvddendhritwdddthttdmeieqptpkkgfkrpqppthnknllarkklrn |
| *Feldmannia species virus* | YP_002154715 | | Mtlklflhdaridnvaspsgsssirdttlvqifavaedgrptyvqvknfrpwfyvdlnefftfaqfksvvaeqywarnvvscelvarkrfigfadgksfdyvlmtftglipmycsrkylrtlkvhlhedsvdpllkffhssgikpssyfemdgfvvwngqgkthcsreyyvavqnlrpsadsgspppipmcsydiessgldpasdyvfqvslcfgflgedldsrsaisdsfvicvgdvesvegtpilcvqnelqllkkfreivverqvcilvgynsyqfdgqflykravdtynyqdfckigflrndkaslktkvlessalgknelsqfvipgrvefdalmtvrrnhklgsykldsvcrhffggkkddvsyeyilsaceskdpkklgviakyclqdawltlrlvsslkdvynglemsklcvvplsyiesrgqqikclslildrvhgefvcnkasrvlpggvkfqgatvidatkgfhnkdpvvcldfaslypsiirwknlcyttyldsdefanipgvhyerfeispgvyetfatrpghkgilsaieedlgearrqtkaamkvekdskklqllnskqlaqkvtmnslygfcgtvngclplvaiaaavtctgrsmikttadfirtemggtviygdtdsvmctfpapqtvrgqgkrallghayamglsaeekslslfghpvkleyekiyfpflliskkryacmsydrpdsepkmstsglvtvrrdnakvvrdcangvisilmegrgqgdvveyvktvlsklenseigvedltisnelkkhpdqyatpsahsvlagklnaraknqklyreivrpvveqggvpslgtaygvlegvrrkfsfdqrrdvsyeeflrdlangrvaeklkgsaprvseceslvgsteakliqegirtekilndmyrefsvfdrmyweaqslgsrvpyvivrgngsvnersedprfvemadhlridtkyyidqqlknpilgiveafpdgraslervfrefsrradnankgrreitsffersvrskv |
| *Heterocapsa circularisquama virus* | AB505427 | | mtrqkinhsicnnlknmpmidvenfkkkklfiieisqktceetmnyilsitcldnngnkyfydvtgiipcfdieyneivaneikkyekilnsnailntrngnivfegvhlkpfnyysnelithirlnfkninnyrtvnnilnknniikeyirnnetntnyynkaiqynnlinvekipektllmtwdietysydktripdglvesddcflicgtfhnynsdkilssfaistintetnvignrsiythtihklifnninnndinnkiynnitqflgiinypnltlkicknekesikcfmkmikeikpdiitgfndhtydwvyiknkienyytdltrdfyecfndskyytltndikpfisssvkisadiglmdinypkcnyaifidtrvefrkiypkdiesnlnyylrkmnlnskedlpykklfqiyenkdedgmllatlycltdayrcqellvkkqiinehynmaymtgitlknsicrangfkvfnyllkdgikenysfiynkfekdensteytggyvadptyglnidcpvigldfaslypniqrtlnlgpdtlirdfdipkylnsnipirkicdkyvvdhnekenlksimvkiltnlfdqrviikkkmlmykfdkekysnvdqyklhltlclfksyklnnkkinnnifdylginvlndvindldqkqkavkvlmnsfygllgsptsplyckfiaetitktgrtmliqvrnyvqnknydvhysdtdsvyvscnkhifndieskfinneitekelfykkiqitkdeikvllndinkylkdtyvysyikmayeevlypvffiskkmyfgvehmetinyddidnflfmrgyspvrrnttqltkniivdtvikqlfslntfsiyhktkkidifeiiknivfqivnnfknktypieyfikndryspgkknirintfvdklrqrfdslsndelkrkytppepyerfkyvyidksnkilyngsiekidglgnvmeypcylddfnsnlhykkyfesdlanelgclihpkdgkkyiiklfelfidnidnkyelnniknidydtlihkkndirtiklkqfrsdakiksiefinikkdllvnypllfkydiisnpytfinninndiskinfdfinqrksdlykkcykneyykpnlneltnalniqynilyeelddnirlycdlisnelnikieehcnnketivdydidksfqnivsfidvnkhsfdnleditdkyynivykikkqesysefikklkkh |
| *Heterosigma akashiwo virus* | BAE06251 | | meiiecqvfsfyptdflnnrnftvfafgrkedgssvgieivdfkpfmylhvperqqkfwtmnntedlrktlvteheikelrnintvmkkrlfpytnkdrelflmlefntewgirkcsmslrenyiyknfdvyesnispmlrlmhmreilpsgwvriknydqnnttkcdtnirinfmdiigferddiapckissfdiecmsfdaytqnqsifpsyerendtisqigmatwsygnneevlkrlytlgnaaeptdqtidiiqcdnegeliirwfhyiaeidpdiitgynifgfdweyikgrvdflgiedeilsvasridklksrfmtkelnssafgdnefkflempgriefdffsyikrehklesykldnvayhftkqkkhdvkpmdifiklmgtpedvrevaeycvqdtfliielmkklcvipnliemskvtrvpfeylilrgqqikvfsqifyeamkeniviptnilklqgkkdrneehekytgatvltansgcyfdcvsgldfaslypsimiaynmcyttlvlnerelpreakietiewennrhrfvqnkegllpkilkklwitrkstkrlmnetenkemktilngkqlaikvsmnsvygfcgvmrgilpcvaiassvttkgrqmiehtqnmvkqlypdakviygdsvtketplmlrtmetcgnhkhevisienvftdnmrsidmysiigekehvmlsrneeiwtgenwsriirvirhktqkkiygvltengyvevtedhslissdyellkpkncivketqllqsfpdivenstiennmidipkgqpcrltvfgqvsamiiytylkrknysitlnvcnvnsnkfyisfmerprfkntkkniikkiffirntdneeyvydvetedgifhagigeiivkntdsvyvnfpstnndmqkvfdisieaaeaisktfpqpielefekvmypfilftkkryasliwtrvdkpdkidfkgiqvvrrdncsyvreslttiyncllyernvdkclgitdkiiddllkgrvpiekltvskslksnyksktmphfllaekmkqrdpmnyprpgervpyvfientearlqgekaenpeyakengliidtlyyldhqmkkplaelfnivlgegkynlyknhmgfikmkknhqererlreinrkkgqkelnlkwfskkk |
| *Micromonas pusilla virus 12T* | YP_007676285 | | Mvvfqaltweardeevpgdddgpvgehlisifgktedgksvcvttafepylyiklpeikyakeiyakikdsctgynvveskdiwgfqnnqkflfmrvtfsnlalrrktdyflkkpmilsngpfplrvyesnldpilrmmhrtgiqstgwldtgsdcvysdlahvdidlfcndwetlkpvkrddvapfvvasidiesnsstgkfpdadvdgdacfqiamslckmgsdesydktcfcfkktdphlegcniysydtelemleafrtymikedidvmtgwnifgfdleyiykraiknecsdsffnlgklkkyetyqgpkqktngsgmvykrlsssalgdnmlkllpmpgrfifdlfhevkkgykldsyklnnvsklylgdqkidmppremfarfaegdpvklrevaeycikdtllphrltkklcillnllemakatwvpisflvergqqikvfsqltkkarelgfmvptirygalppepyegatvleaqggayytpitaldfealypsimmahnlcystfvmdekrygniegityekfelngrtykfaqdvpsllpailselkefrkqakrdmaaatgfmkeiyngkqlaykismnsiygftgagkgilpcvpiastttykgrsmieetknyveknfpgakvrygdtdsvmvefdvggrtgveaieysweigeraadecsalfkkpnnlelekvyhpyflyskkryaaklwtkgkdgnmnmdyidikglqvvrrdntkfvrevckdlldvvmessdpepakqlalerainllegdvsneklvlsqqlgdsyknnnlshvkvrdkmrerkpgsepqsgdrvpyilvktdnprakayekaedpvfieennipidyhhyftnkflnpicdlleplvkdpkneifgeliaqhkpppkkrepalsgmkkeqlieeckklnletdgkvadlrqrikttredkvsidhlfknyd |
| *Only Syngen Nebraska Virus 5* | YP_009325633 | | Msmeifptdwrcedvtpdkgeaffrinifgktaegktvcvqtkftpyfllevpdswsiartnlfitetamkydavrpmclptrrknmwgfdggklrnlvqfvfktqaqmrkakyrlkdqyqlyessvdpiirvfhlrninpadwinisksypaqtrisnsdievetsfqhlgpsdnksvppliiaswdietyskdrkfpladnrtdyciqiattfqkygepepyrrtvvcykqtapvegveiiscleeadvmntwmkilqeektdvsigyntwqydlryvhgrsqmcvddmtgedkvklgnlgrllsgggdvverdlssnafgqnkfflldmpgvmqidllqwfrknrnlesyslnnvsklylgdqkndlpamqifekfegnaddraviaayaakdtdlplkllkkmailedltemanavkvpvdyinfrgqqirafsclvgkarqmnyaipddkawategkyegatvldakkgayftpiaaldfaslypsiirahnmspetlvmdkrfenipgveyyeietglgtfkyaqkndetgdgqgvvpallddlakfrklakknmaeakrngdefkealydaqqrsfkvvmnsvygflgaskgfipcvpiaasvtatgrkmiehtakravellpgsevvygdtdsvmvkmklpddkvhdmdeqfkmakwlageitkdfrapndlevslknserglynfviynpdftflnipgrpvpccssrksttrtsciarkgmlrlslrsqmkkvrltskvlrlmekfmmsktlktgyknecqphlhvankiyertgfpvpsgarvpfvyiedkynpdikqsykaedptfakdndlivdrlfyiehqllkpicslfepllddpekeifghslikekiddlkntfkadlkvakrvkknkannqkeitsffkkk |
| *Organic Lake Phycodnavirus 1* | ADX06143 | | mygldehrttysimvhnftpfvyikvsndwnkhktdefiehlkqhpnksiaygskdivsyewvkkkslygfdankyynfiyiscknmsfvyklrslyyekdtqqlnkgylyknaytkiyecmippllrffhiqnispsgwievkrfsrnkmkkthmdvdincdykniypldkeimvpykicsfdieansshgdfpeaakdykkvaydivyylehqnvqdydyilrellenvfgfkdtllidkcyveqsytyeqfeshmellfkrkiatnyvvanklkeifskddeddeessyvklkkinmndvltmlkditidkpnkivhltnvldetfpqlkgdqvtfigstfvnygeekpylnhciclndshnigdnvlecystekdvlcawsrlimkedpdiiigynifgfdypfmyeranqlncmdefmvlgrnkeqstklfetsivlasgpydlkmlpmsgrlqidlytymrkefnlpsykldyvssylicdkviryenneegqsriysknmkglerlhyvhfeihnhsselyldgkkfqiielyddgflineeldihekfswglskddvspkdifemtkkgphdrgiiakyciqdcnlvhqifqkvdvlttfsemsklcsvpiqflvlrgqgikltsyiskkcrekdtlmpliskgndvdayegaivlepkcglylnnpvacvdysslypssiisenishdskvwtkefdlndhikkdekgrekmtgikdehsnfiydnlpeyeyvdiqydtfeyvrntptsaatkqltgykicryaqfpdnkkailpsilqellaarkatkkqmqketdpfkknildkrqlsikitanslygqtgaktssfyemdvaasttsvgrtlliyakeiienvygntivdtkygkmksnaeyiygdtdsvfftfnfkdlknntlpdkqlldmtievakeaghlctsflkephdleyektfmpfcllskkryvgmlyeddinscsrksmgivlkrrdnapivkdvyggiidilmkekdieksilfldkmleniideniimdklvvtkslrsfyknptriahcvlanrmgardqkpspgdripfvyiknenkqlqgdkietpdfiksegleidyvfyitnqimkpiiqiyslvlndmkcfqrrkpsfiqevetlsknefdtdkrnkkiqsmkekeverllfkkyisfypeemdgmsyqyrtafdvckklgknekeivnailkhkknkk |
| *Organic Lake Phycodnavirus 2* | ADX06483 | | mtlqikmidfqvsemkdefkiqmygldehrktysitvnhfnpfvyilvpniwsksktddfiqhfkdhddktiarsssenivsyelvkkktlygfdankyynfvyiscknmsfiyklkslyydketqqinegylyqnfrtkiyecmippllrffhiqkispsgwieintykkyttkrthmdmdigchykdiisldkddivpykicsfdieassshgdfpeaskdykkvaydmvyfledvpkddyaylleallenvfgfkdtlhidkcypkekytcsqfkihmeklykkkisttketeyklkkyfcnddeeniqvrkvkqadiltmlmddkldnpnkiahmitlldsifpelsgdqvtfigstfvnygqekpylnhciclndtstivdhqiiecydhekdvlcawskliqkedpdiiigynifgfdypfmferanqtncmvefmifgrhkeqsqelietsivlasgpfelkllpmsgrlqidlythmrkeynlpsykldyvssylmsdkvtryennsndtcmiytknmkglelysyvhfeiqnhsselyldgkkfkiiemnvegfviddvldikdsftwglskddvspkeifemtrkgkeergliakyciqdcnlvhqifqkidvmttftemsklcsvpitflvlrgqgikltsyiakkcrekdtlmpliskgnardayegaivlepkcglyldvpvacvdysslypssiisenishdskvwtkefnldhtiklnssgkeciygtkdeqgnfiydnlpdyeyvditydtfayvrktqsaaatkvltgykicryaqfpndekaimpsildellaarkstkkqmknehdpfkqnildkrqlsikitanslygqtgaktstfyemdvaasttaigrklliyakeviegvygnsevdtkygkmktraeyiygdtdsvfftfhfedlngvkikdkqalemtielakeaghlctsflknphdleyektfmpfcllskkryvgmlyeedpekgkrksmgivlkrrdnapivkdvyggiidilmkdknidksidfldtmlqsiidknvimdklvitkslrsfyknpnqiahcvlanrigirdpgnkpapgdripfvyiqttgkklqgerietpqfiqdeqlkidygfyisnqimkpiiqiyslvlydmtkfnrrkrsfiqeiktieeniedndkkqkkynr |
| *Ostreococcus tauri virus 1* | YP_003213031 | | mvvfqaltwesrdtddehlisifgkteegksvclttaftpyffiklpekidagkirriynildekckdslvaysvmkskdvwgfqnneefvfmkvnfkhlqarrlvdsflrkpldrtpelfnifgvrnvkvyesnldpvlrlmhrtgiqstgwldtgdkcirshlarvdmdlfcndwttlkpvarddiapfvvasvdiecnsstgkfpdadvtgdacfqiaislckfgsdepydktclcykktdpnlegstirsyeteremleafqkylhtkdvdiitgwnifgfdmeyiykraqvnrchyeffnlgklrdteselvikklsssalgdnllkllpmpgrfifdmfhevkkgykldsykldnvsklylgdqkidmapkemfaryreedpvklrevaeycikdtllphrlmkklctllnmvemakatwvpanflvergqqikvfsqltkkarelgfmvptirygaipeepyegatvleaqkgayytpitaldfealypsimmahnlcyssyvmdekrygsvpgityetfnigdrtykfaqdvpsllpailaelkqfrkqakrdmaaatgfmkevyngkqlaykvsmnsvygftgagkgilpcvpiastttskgrsmieetknyveknfpgakvrygdsvtpdtpllirengevkttridslvdlyevrddgkeiaeidaevwtecgftpikqivrhkttknihrvlthtgivdvtedhslllknkemikpsevclgtellhgnsleafgethtdvtpeeakvmgfffgdgscghydgkytwalnnadmtfleemselcpfetrvydtiqssgvyklnavgdvksisvryrslfynehkekvvppcilgaplhivqsfwdgyymadgdkdvhgytrmdikgkegsmgmyilgrrlgynvsmntrtdkpdifrqtwttssqrknpiaikklellgetegyvydlttgshhfhvgpgdlvvhntdsvmvefdvgdrkgeeaiayswevgeraaeecsalfkkpnnlelekvywpyflyskkryaaklwtqgkdgkmhmdyidikglqvvrrdntphvrevckelldvvltssdpgppkelakeraiellsgdvpndklilsqglsdtykvggknvsvtsadsvninqshvqvvtkmrqrkpgsepqsgdrvpylltktqdpkakayekaedpkyveehgvpvdyhyyflnkflnpvcdlldplyenvkeeifgeiinrhkppkppklpalstmkkddliaecqrlgleetgtlailrarlkdarhgsvedlfknyeltqskdess |
| *Ostreococcus tauri virus 2* | YP_004063640 | | Mvvfqaltwesrdtddehlisifgkteegksvclttaftpyffiklpenitapkiqriynildekckdslvaysvmkskdvwgfqnneefaymkvnfkhlqarrlvdsflrkpldrtpelfdifgvrnvkvyesnldpvlrlmhrtgiqstgwldtgdkcirshlarvdldlfcndwttlkpvvrddiapfvvasvdiecnsstgkfpdanvpgdacfqiaislckfgsdepydktclcykktdpnlegsnilsydteremleafqkylhksdvdiitgwnifgfdmeyiykraqvnrchyeffnlgklrdteselvikklsssalgdnllkllpmsgrfifdmfhevkkgykldsykldsvsklylgdqkidmapkemfarynegdpvklrevaeycikdtllphrlmkklctllnmvemakatwvpanflvergqqikvfsqltkkarelgfmvptirygaipeepyegatvleaqkgayytpitaldfealypsimmahnlcyssyvmdekkygavpgityetfkvgdrtykfaqdvpsllpaillelkqfrkqakrdmaaatgfmkevyngkqlaykismnsvygftgagkgilpcvpiastttckgramieetknyveknfpgakvrygdtdsvmvefdvgdrkgedaiayswevgeraaeecsalfkkpnnlelekvywpyflyskkryaaklwtqgkdgkmhmdyidikglqvvrrdntphvrevckelldvvltssdpgppkelakeraiellsgdvpnhklilsqglsdtykvggknvsvtskesvninqshvqvvtkmrqrkpgsepqsgdrvpylltktqdskakayekaedpkyveehgvpvdyhyyflnkflnpvcdlldplfenvkdeifgeiinqhkppkpkrepalstmkkddliaeckrlsldetgtlavlrarlkearqgsvedlfkkyeltqsknessredhads |
| *Paramecium bursaria Chlorella virus 1* | AAC00532 | | mtditifptdwraedvvpdkgesffrinifgktaegktvcvqtkftpyfllevpeswspartnlfitetamkydavrpmclstkrknmwgfdggkmrnmvqfvfktqaqlrkakyrlkdqyqiyessvdpiirvfhlrninpadwirvskaypaqtrisnsdievetsfqhlgpvedktvpplviaswdietyskdrkfplaenptdyciqiattfqkygepepyrrvvvcykqtapvegveiiscleesdvmntwmkilqdektdvsigyntwqydlryvhgrtqmcvddmtgedkvklsnlgrllsgggevverdlssnafgqnkfflldmpgvmqidllqwfrknrnlesyslnnvsklylgdqkndlpamqifekfegnaedraiiaayaakdtdlplkllkkmailedltemanavkvpvdyinfrgqqirafsclvgkarqmnyaipddkawategkyegatvldakkgayftpiaaldfaslypsiirahnmspetlvmekrfenvpgveyyeietglgkfkyaqkndetgegqgvvpallddlakfrklakkhmaeakrngddfkealydaqqrsfkvvmnsvygflgaskgfipcvpiaasvtatgrkmiehtakravellpgseviygdtdsvmvkmklpddkvhdmdeqfkmakwlageitkdfrapndlefekiyypyilyskkryaavkfeepdekgkvdvkglalvrrdfspitrdilkesldtilykkdtptavsetlerirkvldneypmekfmmskllktgyknecqphlhvankiyertgfpvpsgarvpfvyiedkknpdikqsfkaedptfaqdnglivdrlfyiehqllkpicslfepllddpekeifghrlikekienlknvfkadlkvakrvkkniannqreitsffkkk |
| *Paramecium bursaria Chlorella virus AR158* | YP_001498312 | | mteltffptdwrsedvepdkgepyfrinifgktmdgkticvrakftpfflletpeswsaartnlfitetamkydairpsclptkrknmwgydggkmrpmvqfvfktlsqmrkakyrlkneyqiyessvdpiirifhlrninpadwvhvskafpvetrisnsdievetsfqhlgpsdvkevppliiaswdietyskdrkfplaenstdyciqiattfqkygepepyrrvvvcykqtapvegveiiscaeeadvmntwmkilqdektdisigyntwqydlryihgrsmmcvdditgednvrlknlgrllvgggevierdlssnafgqnkfflldmpgvmqidllqwfrknrnlesyslnnvsklylgdqkndlpamqifekfeggaddraiiaayaakdtdlplkllkkmaileditemanavkvpvdyinfrgqqvrafsclvgkarqmnyaipddkmwtvdgkyegatvldakkgayftpiaaldfaslypsiirahnmspetlvmdkrfenlpgieyyeietglgtfkyaqkndetgegqgvvpallddlakfrkqakkhmaeakknddefrealydaqqrsfkvvmnsvygflgasrgfipcvpiaasvtatgrkmiehtakrvtellpgseviygdtdsvmirmklpddkihdmdeqfkmakwlageitkdfkapndlefekiyypyilyskkryaaikfedpdekgkvdvkglalvrrdfspitreilkesldtilfkkdtptavtetvecirkvldneypmekftmsktlktgyknecqphlhvsnkifertgfpvpsgarvpfvyiedkknldtkqsfraedptfaqendlivdrlfyiehqlmkpicslfepllddpeteifghplikgkidelkstfkadlrdakrtkkniannqreitsffkkk |
| *Paramecium bursaria Chlorella virus FR483* | YP_000425655 | | msfdpgiistkracdtkfhniqyynlssdmtsltvfptdwrsgdegeqfrinlfgktpdgktacirirftpvfllelpaawspsrqklfitetaikygaikdmclpvkkksmwgfdggvmrnlaqfafptlekmrkakyglkrdyqiyesnvdpivrlfhirkinpagwvqikqsypvmtrisrsdievdcnfttvsvselttppplviaswdietyskerkfplssnptdyvtqiatsfqrygeeepyrrvvvcfkdtgkvdgveivscseeqdminawmtivseektdvligynvfqydwkyvsgraqmlvddasaddtvfvdtlgrllegggavverelasnafgqnffyyldtpgviqldllqwmrknrnlesyslnnvsklylgdqkddlpamqifekfeggpedraviakyaaqdtllplkllsklaifeditemanavkvpvdwigfrgqqvrafsclfgkaremnyaipddkawaaegkfegatvlepkkgayftpiaaldfaslypsiirahnmspetlvmdaryknlpgveyyeigtgigtfrysqqsqgvvpallddlakfrknakklmaaahkegddfkealydasqrsykvvmnsvygflgaskgflpcvpiaasvtatgrnmidvasrraiellpgseviygdtdsimvkmklpegknqedindhfevakwlageitkeyrapndlefekiyypyilyskkryaaikyedpeekgkvdvkglalvrrdfspitreilkesldtilfakdtptavkdtrekirkvldneypmekfvmsktlktgyknemqphlivankifdrtgfpvpsgarvpfvyvedkdnidakqsmraedpkyamdnglivdrlfyinhqllkpltslfeplvdhpekelfghvdvvgkivalatrhkaelkdtkrvkknkannqieitsffkpktlkl |
| *Parameicum bursaria Chlorella virus NY2A* | AAA88827 | | mteltffptdwrsedvepdkgepyfrinifgktmdgktvcvrakftpfflletpeswsaartnlfitetamkydairpsclptkrknmwgydggkmrpmvqfvfktlsqmrkakyrlkneyqiyessvdpiirifhlrninpadwmhvskafpvetrisnsdievetsfqhlgpsdlkevppliiaswdietyskdrkfplaenpadyciqiattfqkygepepyrrvvvcykqtasvegveiiscaeeadvmntwmtilqdeitdvsigynlwqydlryihgrsmmcvdditgednvrlknlgrllvgggevierdlssnafgqnkfflldmpgvmqidllqwfrknrnlesyslnnvsklylgdqkndlpamqifekfeggaddraiiaayarkdtdlplkllkkmaileditemanavkvpvdyinfrgqqvrafsclvgkarqmnyaipddkmwtvdgkyegatvldakkgayftsiaaldfaslypsiirahnmspetlvmdkrfenlpgieyyeietglgtfkypqkndetgegqgvvpallddlakfrkqakkhmaeakknddefrealydaqqrsykivmnsvygflgasrgfipcvpiaasvtatgrkmiehtakrvtellpgseviygdtdsvmirmklpddkihdmdeqfkmakwlageitkdfkapndlefekiyypyilyskkryaaikfedpdekgkvdvkglalvrrdfspitreilkesldtilfkkdtptavtetvecirkvldneypmekftmsktlktgyknecqphlhvsnkifertgfpvpsgarvpfvyiedkknldtkqsfraedptfaqendlivdrlfyiehqlmkpicslfepllddpeteifghplikgkidelkstfkadlrdakrtkkniannqreitsffkkk |
| *Phaeocystis globosa virus 16T* (Gp I) | YP_008052566 | | mkakptvktksyklydfnvydgfskaenlgkngydkfkdnkkfiiqmfginvagetvslivedfnpfyyikvgddwgesdraefiahiknklgayyedsivasklvkrhklygfddnklhtfikisftntgaynrakkmfyvdstvdgvfkrelipdgylyeetkcylyeanippllklfhiqeispsgwiqiqsdkitkmrqksthcayeyitsykhlkkadkddivkysicsfdieassshgdfpvpikdykklatnileyynemedktkfdvtcfkklidagygyercpdiatvypklkniskeqldnifsnfmlyipakdqsrkdyikeneesdtdsdnddeddkkendgadeaaafhkrhkkvkkyhktanllqiihddkcendtklfelnkaltkfypelegdmvtfigmtflnysekkphtryiivkggcevpekykswvlennvkiiekttekgvlleftkimtlenphivtgyningfdfdfmfkrskeigcvedflklsknidevcmtkdwktgemeiaknkivlasgeynlsfinmpgriivdmcvvfrreytlssnkldyvssyfisdsvkkidvdkennqtriysknltgltvgcyvkfdevshstnnykkgqkyeildinldtasflidsaeeldlkkykinwglakddvsvqeifelanksdidrftvgkycigdcdnviwllikvdiitdkvemsnlcdvplnfllqrgqgiklqsyvskkcgekntlmpiveknlndggyegahvfnpktglyledpvacvdysslypssmisenlshdskvwtkeydlsdnlihstgekdadenfiydnlpnytyvdvkydtyeylrktpkaaekktvvgykicrfaqfpkgkaimpailedllsarkatkklmgkeedpfkqniydkrqlsikvtanslygqcgartsafyekdvaasctaigrkllfygkdviegcynnveitlsdgvkvvtkaecvygdtdsvffkfnlktpegkriinkqaliytielakqagelatkflkkphdleyektfwpfnllskkrydgmlyendpeycklksmgnvlkrrdnapivkdiyggvvgilmkdkslpksikfvkesvqnmidekypiekllvtkalrgyyknpkqiahkvladrigvreqgnkpgagdrmnyayiknpnkkalqgekietpefikdnelkldyghyitnqimkpllqlyaleleniqefkdkqfnikeyntdkkvilweeeiiklkekwpdpekyvkkyeelrckevkalifdkylkglk |
| *Phaeocystis pouchetii virus* | ABU23718 | | Mttktviktksyklydfnvydgfskaenlgkngydkfkdnkkfiiqmfgintagqtasiivedfnpfyyikvgddwtesdrcefvshikgklgvyyedsivasklvkrhklygfddnklhtfikisftntgaynrakkmfyidsnvdgvfkrellpdgylyeetkcylyeanippllklfhiqeispsgwvqiqtdkitkmrqksthcayeyitsykhlkkadkddivkysicsfdieassshgdfpvpikdykklatnileyynemedktkfdvtcfkkhinagygyercpdiatvypklknisneqldnifsnfmlyipakdqsrkdyikeneesdsesdnddeedkkendgadeaaafhkrhkkvkkynktanilqiiqddkcehdtklfelnkaltkfypelegdmvtfigmtflnysdkkphtryiivkggcevpekykswvlennvkiiekttekgvlleftkimtlenphivtgyningfdfdfmfkrskeigctedflklsknidevcmtkdwktgemeiaknkivlasgeynlsfvnmpgriivdmcvvfrreytlssnkldyvssyfisdsvkkidvdkennqtriysknltgltvgcyvkfdevshstnnykkgqkfeildinldtasflidsaeeldlkkykinwglakddvsvqeifelanksdidrftvgkyclgdcdnviwllikvdiitdkvemsnlcdvplnfllqrgqgiklqsyvskkcgekntlmpiveknlndggyegahvfnpktglyledpvacvdysslypssmisenlshdskvwtkeydlsnnlihstgekdadenfiydnlpnytyvdvkydtyeylrktpkaaekktvvgykicrfaqfpkgkaimpailedllsarkatkklmgkeedpfkqniydkrqlsikvtanslygqcgartsafyekdvaasctaigrkllfygkdviegcynnveitlsdgvkvvtkaecvygdtdsvffkfnlktpegkriinkqaliytielakqagelatkflkkptrfrvrenilafqsiiqeki |
| *Prymnesium kappa virus* | AIS68742.1 | | Vlleftkviqtenphiitgynitgfdfefmfkrsqelncsneflklsrnigeicinkdwrtgkedietskivlasgeynlkfvkmpgriiidmytvfrkeyilsgykldytssyfisdtinnieinyqnnttkifsknltgltvgcyvkfeeisysvnnykkgk |
| *Pyramimonas orientalis virus* | ABU23717 | | mnfrdhsisldesknacfqvidwyhfdytnedsnesqyiikmfgvteegyslcvnvtdfqphfyissktkdkftqtelddleeyiinklpynfknslsvkqvrkksiwgftnnvykqyiklsfqnimsmyitrkmlqyrikvgrvqvqfdlnesnidpflrfihiqnikpggwisideyttdvddeleskcqinittscenvqpldcnkvapvnimsfdiectsssgdfpvpiktykktaeeisdlynsfksdshhdgfipalykcfmgiytdekefmndcgtycnknllecntnnikfakvypkkkkidletifqklqgytdtivdilknrskhtvfqeendglepsvvnqlevllnsflpplkgdpiiqigstihqygstkcsyknvitldtcndipgvdvitcktetalikewcklihrvdpdimtgynifgfdfdyiykralelgcekyvlgcsrleghkskfkekmlassalgenllkyvemegrvfvdlmkvvqreynldsykldnvashfisgkvkthnkttlhldsaaginvgdyiklnntykcmvlsvdsniividteldekivtwglakddvspkeifacqkgtsadrakiakycvqdcalcnmliiklevfannmgmanvclvplsyifmrgqgikifslvakqcrddnfiiplikfdqdseevegyegaivldpipgiyveapisvmdyaslypssmisenishdsivldkkydnlpgveyvdvtydiftgvgdkktkvdektcryaqfknnekgvlprilmkllsqrkstrkqilhktvttndnrsftglvdenddsvtikttdnntitlsrneilssvdtyndfmkaildglqlaykitanslygqvgartspiymkelaasttatgrnlimkakefmetnygadvvygdtdsifvdfkvkekyglldkdalqksidisvkasdafkkelkaphdleyektffpfiilskkkyvgnlyehdvnkykqksmgivlkrrdnanivkivyggiidillnrqnisfainflkeslrklvngefhmddlvitktlrttykdptriahkvladrmrnrdpgsapqssdrvpyayiehdiknksllqgdkiehpsyikennvkidyifyitnqlmkpicqlmalalfqipksskpeiyerklksltldyegnkkraidkvsdlkqkeiqkllfdetlvhlnnkrmgnreileffsvtpv |
| *Tetraselmis Virus 1* | AUF82649 | | mkfqivdwevsdvpeteipecfvtdthsrfqddeddaprylytvivygrtkegksvclkltqfqpyfylkvdgikdytphlsnisrhiisncfhlkreiqhivigkkcdfygfsanekkecvivtfdswramrsvasklrssafkyqnklitlyetrveplmrllhsreikstgwveiedskiefenwgatdicgttsyknvdplevddiapmlmasfdiectvesdifgdfpvaiksyrriannikdtleiamkydldpykakeliqivifytlgmkdvegrfadtvnwmnrnnascltqrvlgsvrtketlsttalynylyiivddlfsyatgdikyedksqpsgpveqidsllsktlpplkgdpviqigvtcntigntecsskwisvlnscdhipgvevesyeteeevimafaryirksspdlitgynifgfdmkylhertlelgisdkflrtisrlnthpakfvekrlsssalgdnvfwmldipgivsldlmkvvmrdhklesykldnvaqhftgekkrdvppkdifrlqngtskdrsvvadycvqdcalcnvlleklkvvvnaiamasmshvpisyiflrgqgvkilslvsyycrkrdmiikdlpkkleepdedaplwkrnryrqeaeaqefmekpddievqgaivlepdvgfyvddpvavcdyaslypssilshgispdawvkddsmrkhpdytykdvsyeiyegkgddkkvvgmetltfaikknvdvydpntlsiipaierillmgrksirkkakhkkvvtkdgteyvgdyseldgdrikingvvlknddvvsvnnfynqfmqdvldgqqlsykllansvygqlgartsdirdmklaacvtsvgrsliisakdfihknggtvvygdtdsvfctfpvydpvtgerlkgkdalpyviektqyigkeftktilppphdleyektfwpllllakkkyvgnmyeedpekfklkymgvvlkrrdnapivrrvistlldvllneidvtkaldnmlkviddvvnanvpieelvitktlraeykdrsriahavlaqrigerdpgnapqindripmvhirvpakkgekilqgdkvedpeyvqsaglepdmeyylenqimnptiqmlaallekipgynkpigyyerktdeytlqykndtsrnlteetirkkvreaidkirqeevrklvfepklakirnkykgqldlttmgfffkm |
| *Yellowstone Lake Phycodnavirus 1* | YP_009174732 | | Mttfqavawegsdhedgkfviriygrqadgksvalgttfrpyfyvklrvqhnfadfaalirkkfqpteikevrakdlwgfqnnllsrfariefdtmrqmrfcayglrksdtefgklklyetnidpvlrfmhvtgirstgwltcaatepdydttcdinlwapehtnikpvdrddvaplkimsfdiecysksgnfpdpmktedcvfqigmttrkfgsdepmerkclcfkntagpdaesfdtekkllqafdkyliktdpdiitgwnifgfdleflqvravknglaptwgrfkdspielvtknlsssalgnnmlkmvpmrgryvfdlfqdvkrehklesyslnnvskhflkdqkndmpvkeifsrfaegdparlgevaeyclkdtelphklldklcqiqnlvemakacwvplaflsergqqikvfsqmaykarelnfiiptfergpaldddkyqgatvldaqtgayyspitaldfaslypsimcahnlcystlvmdpkfdnlpgvtyeqfgphrfaqnvpsllpviltdlkayrkkakklmaqaegtpmeaiyngqqlaykismnsiygftgaskgmlplvaiastvtmrgrqmieetktyveenfpgakvrygdtdsvmvefdvqgrkgqeaidyswqlgeqaaeqctklfkapndlelekvycpyflyskkryaakmyeknklgeiafkkidvkglqvvrrdscpyvretlkqllnmvlesddpkpavnfakqsakdlkaglvpieklllskqlasdykvkmphvevrdkirarapgsepqqgdrvqfvivegrgrmfekaedpewvktnglkidyeyyfghqlkkpvcdlleplvggdpekvifapkvktmtdffslrpapkve |
| *Yellowstone Lake Phycodnavirus 2* | YP_009174598 | | mqsqavawegddvddryivriygrcedgrsicvstpfepyffikirpshkfavlknalsrhfydlteiqeilakdlwgfrnglrerfvkltfktiksmricaamidrmkerddrwedlkafgqlkiyesnmdpvlrfmhvsgvrstgwfevaggtpdystscnvnlwvedyknitpvdrddvaplrvmsfdiecysstgefpnpnttrdvvfqigmttrtfgsdepmvrkclclkqtdaadcesfeterallerfekyltevdpdimtgwnifgfdleylqvrsvlcglaptwgrfkdspielvtknlsssalgnnllklvpmrgryvfdffqdvkrehklesyslnnvskhflkdqkndmpvkeifsrfaegdpkrlgevaeyciqdtvlphklldklcqlqnqiemakacwvplsflsergqqikvfsqmaykarqlgfiipvfkkplvsgpddgyqgatvldaqtgayygpitaldfaslypsimcahnlcyssmvmdpqfdnlegveyeqfgqfrfaqnvpsllpviltdlkafrkkakklmaaaegtpmeavyngqqlaykismnsiygftgaskgmlplvaiastvtmrgrqmieetknyveanfpgahvrygdtdsvmvefdvqgrkgqeaidyswqqgeqaaeqctklfkapndlelekvycpyflyskkryaakmyekgrdgavvfkkidvkglqvvrrdscpyvretlkkllemilesddprppvefakqaakaltngevptdkllmskqlaanykvrmphvevrdkirkrapgsepqqgdrvafvivegpknakmfekaedpewvlekkikidyqyyftnqlkkpvcdllepllgrdteklifqpkvrtitqfftpk |
